# Female Gaming, Gaming Addiction, and the Role of Women Within Gaming Culture: A Narrative Literature Review

**DOI:** 10.3389/fpsyt.2019.00454

**Published:** 2019-07-10

**Authors:** Olatz Lopez-Fernandez, A. Jess Williams, Mark D. Griffiths, Daria J. Kuss

**Affiliations:** ^1^International Gaming Research Unit, Department of Psychology, Nottingham Trent University, Nottingham, United Kingdom; ^2^Turning Point, Eastern Health and Clinical School, Monash University, Melbourne, VIC, Australia; ^3^Institute for Mental Health, School of Psychology, University of Birmingham, Birmingham, United Kingdom

**Keywords:** internet addiction, internet gaming disorder, gaming disorder, behavioral addiction, female gaming, gaming culture, narrative review

## Abstract

Research investigating female gaming has begun to emerge despite gaming being traditionally more popular with males. Research in the 21st century has drawn attention to the role of women in culture, society, and technology, and female gaming is one of the growing phenomena not to have been researched in depth. The aim of the present paper was to review female gaming (i.e., the role of females within video game culture) and identify any associated psychopathological symptomatology. The review adapted the Sample, Phenomenon of Interest, Design, Evaluation, Research (SPIDER) model in conducting a narrative literature review. A search of three scientific electronic databases yielded 49 papers for further evaluation. From a methodological perspective, studies had to fulfill the following criteria to be included: i) published between the years 2000 and 2018; ii) assessed female gaming or the female position within gaming culture, iii) contained quantitative, qualitative, or mixed methods approaches to produce empirical data or discuss theoretical implications through reviews, iv) be retrievable as a full-text peer-reviewed journal paper, and v) published in English, German, Polish, Spanish, Italian, Portuguese, or French. Four categories emerged from the papers: i) the benefits of female gaming, ii) why women might play video games less than men, iii) perceptions and realities of female characters within video games, and iv) women’s position in gaming culture. The main findings showed playing video games has benefits for women in terms of enhancing cognitive, social, and physical abilities. However, they are less encouraged to play video games due to negative expectations based on gender and/or experiences during game play. Video games are associated with stereotypical male characteristics, such as being overly aggressive, and frequently contain sexualized content. Female gamers appear to require coping strategies to handle online harassment. Females look for different things in video games, which are not often included in game designs thereby limiting their abilities. For instance, female avatar representation—which is exaggerated and hypersexualized—can prompt social comparisons and lead to feelings of decreased self-esteem, depression, and other impacts on well-being. Overall, there are still obstacles for women playing video games even though they comprise half of the gaming population.

## Introduction

Over the past two decades, the number of female video game players has increased, and females today make up half of the gaming population according to both the Entertainment Software Association (ESA) (1) and the Interactive Software Federation of Europe (ISFE) (2). Simultaneously, research on addictive internet use has proliferated, and gaming disorder was recently recognized as a new mental health disorder (and a behavioral addiction) by the World Health Organization (3). Thus, gaming addiction is now officially a new psychopathology that has emerged as a consequence of the development and increasing popularity of video games and online technologies during the 21st century.

Despite the growing female gaming population, almost all research on gaming addiction is focused on male gamers. Currently, there is no agreement on the prevalence of gaming addiction due to its conceptualization and methodological problems within the research that has been conducted to date. This is because most research i) comprises surveys using non-representative samples (i.e., instead of using randomized samples, or other methodological approaches, such as classic experiments or mixed methods research); ii) uses different scales to assess problematic gaming [e.g., Problem Video Game Playing Questionnaire (4) for offline and online video gaming, Internet Gaming Disorder Scale–Short-Form (5)]; iii) uses scales and screens based on different addiction criteria (e.g., substance use disorder criteria, gambling disorder criteria); iv) uses different cutoff scores even when using the same instrument; v) utilizes different conceptualizations of gaming [e.g., problematic gaming, addictive gaming, internet gaming disorder (IGD), online gaming disorder, offline gaming disorder]; vi) assesses gaming without taking into account the various technologies and devices (e.g., computers, consoles, smartphones, and tablets); and vii) does not tend to take into account the different game genres played [e.g., massively multiplayer online role-playing games (MMORPGs), multiplayer online battle arena (MOBA) games, etc.].

For these reasons, the estimated prevalence rates of disordered gaming have been reported to range from 0.2% up to 34% (6), which is highly variable for a disorder. However, among samples using nationally representative data, the range is much smaller (up to 9% at most) (6). Higher prevalence rates are reported in Asian countries, especially in young adult males (7). However, very little attention has been paid to the societal and cultural parameters associated with gaming, even in cross-cultural studies. These tend to have a robust psychometric approach that usually guarantees measurement invariance across languages (8, 9) and focus on individual factors (10, 11). However, these recent studies suggest an integrative biocultural approach (i.e., to distinguish universal as opposed to culturally contingent dimensions of human suffering) as well as addressing socio-cultural factors and how these impact on mental health (in general) and problem gaming as a potential form of behavioral addiction (more specifically).

During the past decade, a few empirical studies have specifically researched female gaming (12–14). Through interviews and online diaries, Lewis and Griffiths (12), as well as McLean and Griffiths (15), highlighted that women usually play casual games typically for shorter periods compared with men. They take on their own female roles as gamers and have their own i) gaming experience and habits, ii) game motivations and choices, iii) technology preferences, and iv) gamer identity. In summary, this research has found that female casual gamers report i) peripheral knowledge from gaming [i.e., technical knowledge, games knowledge in First-Person Shooter (FPS) and MMORPGs]; ii) gaming as a domestic life priority (i.e., gaming as an activity influenced by shared vs. non-shared leisure pursuit, unsympathetic partner, facilitating social behavior; e.g., *Wii Walk It Out*); iii) gaming as a personal preoccupation (e.g., as routine in daily life, to satisfy an emotional need, for competition and/or self-challenge, as an enjoyable waste of time, or as a hobby); and iv) gaming and technology as gendered (i.e., concerns about gendered stereotypes) because female gamers characterize themselves as “tech-savvy” (which goes alongside social perceptions as “geeky guys” because gamer identity appears to be more associated with masculinity). However, it has also been found that women feel comfortable making technology purchases and consider age as a more important factor than gender, which appears to negatively affect older women who do not grow up with technologies and online video games. Finally, female identity is sometimes viewed as vulnerable and may underlie why some female gamers utilize male avatars in-game.

However, from a neuroimaging perspective, Wang and colleagues (14) recently found that females appear to be more vulnerable to online gaming addiction relative to males. The study tried to get a better understanding of sex differences relating to biological mechanisms underlying IGD, a proposed mental health disorder included in the final section of the latest (fifth) *Diagnostic and Statistical Manual of Mental Disorders* (DSM-5) by the American Psychiatric Association (16). They used the structural magnetic resonance imaging technique and detected a group-by-sex interaction. More specifically, they found that male and female IGD participants had increased and reduced cortical thickness, respectively, alongside their right posterior cingulate cortex (PCC) compared to same-sex recreational game players. Contrarily, male and female IGD participants reduced and increased cortical thickness, respectively, in their right PCC. Moreover, only females had negative correlations between cortical thickness and their self-reported cravings and IGD scores. These findings suggest that males and females are differently affected by IGD and that women are more vulnerable than men based on the effects created by IGD in the brain regions examined.

More recent quantitative studies analyzing IGD (17, 18) have found that the prevalence of disordered gaming appears to be more balanced than previous studies. For instance, a recent study (17) found differences between genders when comparing specific problematic internet uses, where the potential at-risk problem online gamers comprised 10.8% of the total sample (i.e., 5.3% males and 5.5% females). Moreover, how gaming preference affects IGD scores across genders has been also observed (18), where IGD was predicted by several variables with gender differences. This included time spent online, gaming motives, and depressive symptoms. For female gamers, IGD predictors included higher time spent online, higher scores on specific gaming motives (i.e., escape and competition), together with significant depressive symptoms, compared with male gamers [e.g., whose IGD predictors were two types of motives to play online video games (i.e., escape and coping), together with higher depressive symptoms than females].

Another gender issue in IGD concerns gaming preference across genders. For example, the respective game genre that individuals play has a different effect on IGD score depending upon the player’s gender, although results are contradictory (e.g., men appear to prefer MMORPGs, while women appear to prefer casual video games) (18). For men, coping is a predictor of IGD, while for women, competition is a predictor, whereas escapism is a predictor of IGD in both genders. However, in a recent quantitative study concerning female gaming, women who played video games also reported spending more time on role-playing games, MMORPGs, FPS games, simulation games, action-adventure games, casual games, and MOBA games. Moreover, achievement and social motivations were predictors of IGD and daily time spent online (19). Thus, the preferred game genre may explain differences between genders in terms of time spent gaming (e.g., especially FPS games, MMORPGs, and MOBA games) and IGD scores (e.g., especially MMOPRGs and MOBA games), at least among female gamers (19).

Clinicians treating gaming addiction have reported that this mental disorder may go unnoticed in females (20) and that women being treated for this problem appear to show differences in the experience of other psychopathologies (including IGD and other addictions) compared with men (21). Nevertheless, problematic and potentially pathological gaming in women has rarely been addressed in either theoretical work or empirical research.

With respect to the scarce literature on female gaming and female gaming culture (22), women are arguably situated outside of video game culture (i.e., they are not part of traditional masculine gaming culture) (18, 19), which results in a low gamer identity profile (and is a reason why women may choose a male avatar while gaming or is more competitive than male gamers), and needs to take into account about how and why female gaming can become stigmatized. Women are often discriminated against by male players, which also discourages women from labeling themselves as gamers (13).

A number of comprehensive narrative reviews have been undertaken regarding the phenomenon of female gaming relating to gaming culture. According to male gender stereotypes, women are not considered as “true” or “hard-core” gamers (where video game skill is viewed as the main defining feature of a “gamer,” e.g., playing more complex and competitive video games on dedicated consoles, identifying with the gaming community, and sometimes engaging in competitive electronic sports where gamers can earn money in international tournaments), mainly because they appear to play more casually and less skillfully compared to their male counterparts (23). However, this depends on how “gamer” is defined and the fact that most professional gamers are male. Furthermore, female players who achieve a high level of skill and competence are invisible and/or actively marginalized and may be problematic in terms of the conceptualization of “female gamer.”

Moreover, the association between representation of women within video games and their well-being has been recently studied (24). Findings showed female gamers report self-objectification and consequently perceive low levels of self-efficacy, which was corroborated by both genders (e.g., female characters are usually subordinate to the male hero, in addition to being objectified and hypersexualized). Nevertheless, solutions to some of the effects of stereotype threat on females’ gaming performance have been demonstrated *via* experiments. Kaye and Pennington (25) examined the impact of stereotype threat on female online gamers’ performance (i.e., situations in which individuals’ performance may be hindered by stereotype-salient cues), and whether manipulating the availability of multiple social identities (i.e., personal self and the self as a product of valued social groups) is established effectively for eliminating these performance decrements. Findings showed that stereotype-threatened females underperformed on the gaming task relative to males in the control condition (e.g., prejudice in online video games), and the intervention of multiple social identities appeared to protect females’ gaming performance from stereotype threat (e.g., *via* more supportive gaming communities through inter-group cooperative tasks).

Thus, two types of harms appear to be associated with female gaming at present: i) the personal harm of potential gaming addiction at an individual and psychopathological level, and ii) the societal harm of stigmatizing female gaming at a community and psychosocial level. To date, few studies have focused on the second type of harm studies, and even fewer have examined gaming behaviors based on individual gamers’ perceptions and potential risk of psychopathology, such as gaming addiction [e.g., IGD (17–19) or Gaming Disorder (5)]. Moreover, almost all studies reported have focused on negative consequences associated with female gaming without assessing female gaming behavior from both positive and negative perspectives at individual and community levels. Consequently, there is a gap in knowledge regarding female gaming from a gender perspective, including its nature, benefits, and potential risks to individual and community health.

In order to overcome the limitations in female gaming research, the aim of this narrative literature review is to provide a comprehensive overview of studies assessing female gaming or the position of women within gaming culture. The present paper includes studies from both an individual perspective and a cultural perspective in order to obtain a more inclusive and contemporary view of gaming behavior in females.

## Methods

### Data Source, Search Strategy, and Research Questions

A narrative review of the literature was undertaken to identify all of the relevant publications concerning female gaming, female gaming addiction, and the position of women within gaming culture. The review adapted the Sample, Phenomenon of Interest, Design, Evaluation, Research (SPIDER) model in conducting a narrative literature review (25). This is an alternative search strategy tool compared to the Population, Intervention, Comparison, Outcome (PICO) model, which is usually used as a systematic search strategy tool intended for quantitative research questions (26). The following research questions where formulated: 1) What is the role of the female gamers in gaming behavior and gaming culture in contemporary society? 2) Which variables have an influence on the role of female gamers at an individual and community level?

Between February and March 2018, a literature search was conducted using the scientific databases *Web of Science* (*WoS*), *PsycInfo*, and *PubMed*. The following search terms were entered with regard to female gaming, specifically, girl* OR woman OR female* OR women AND game* OR gaming AND mobile OR online OR video* OR digital OR MMO* OR MOBA OR virtual OR VR OR AR OR FPS.

### Eligibility Criteria

The SPIDER model structured the search terms and eligibility of criteria (see **Table 1**).

**Table 1:** SPIDER Table of Study Inclusion and Exclusion Criteria.

	Inclusion	Exclusion
Sample	Prioritization was given to female gamers, although other samples including male gamers were included if there was a subsample of women analyzed independently as a specific gender	Female gamers not addressing female gamers issues at an individual or community levels; male gamer samples; or female and male gamer samples where both were analyzed together as a unique gender
Phenomenon of Interest	Studies about female gaming or females in gaming culture	Studies examining other related themes (e.g., male gamers, industry professionals, etc). Studies which do not specifically consider gaming or gaming culture
Design	Theoretical (e.g., reviews) or empirical peer-reviewed papers with all methodological approaches (e.g., experiments, survey, qualitative or mixed-methods)	Non-peer reviewed papers (e.g., gray literature, book chapters, conference proceedings, PhD theses, etc).
Evaluation	Synthesis, quantitative analysis, qualitative or mixed methods analyses of benefits of female gaming, why women play video games, and female characters within video games; and women’s role in gaming culture.	Any paper not addressing the topic included in the research questions (e.g., women learning electroacoustic composition)
Research Type	Peer-reviewed journal articles published between the years 2000 and 2018, with full text available in English, German, Polish, Spanish, Italian, Portuguese, or French.	Peer-reviewed papers published in the 20th century. Gray literature (e.g., conference papers, reports, thesis, dissertations), protocols, editorials, opinion pieces, etc).

### Study Selection and Data Extraction

**Figure 1:**
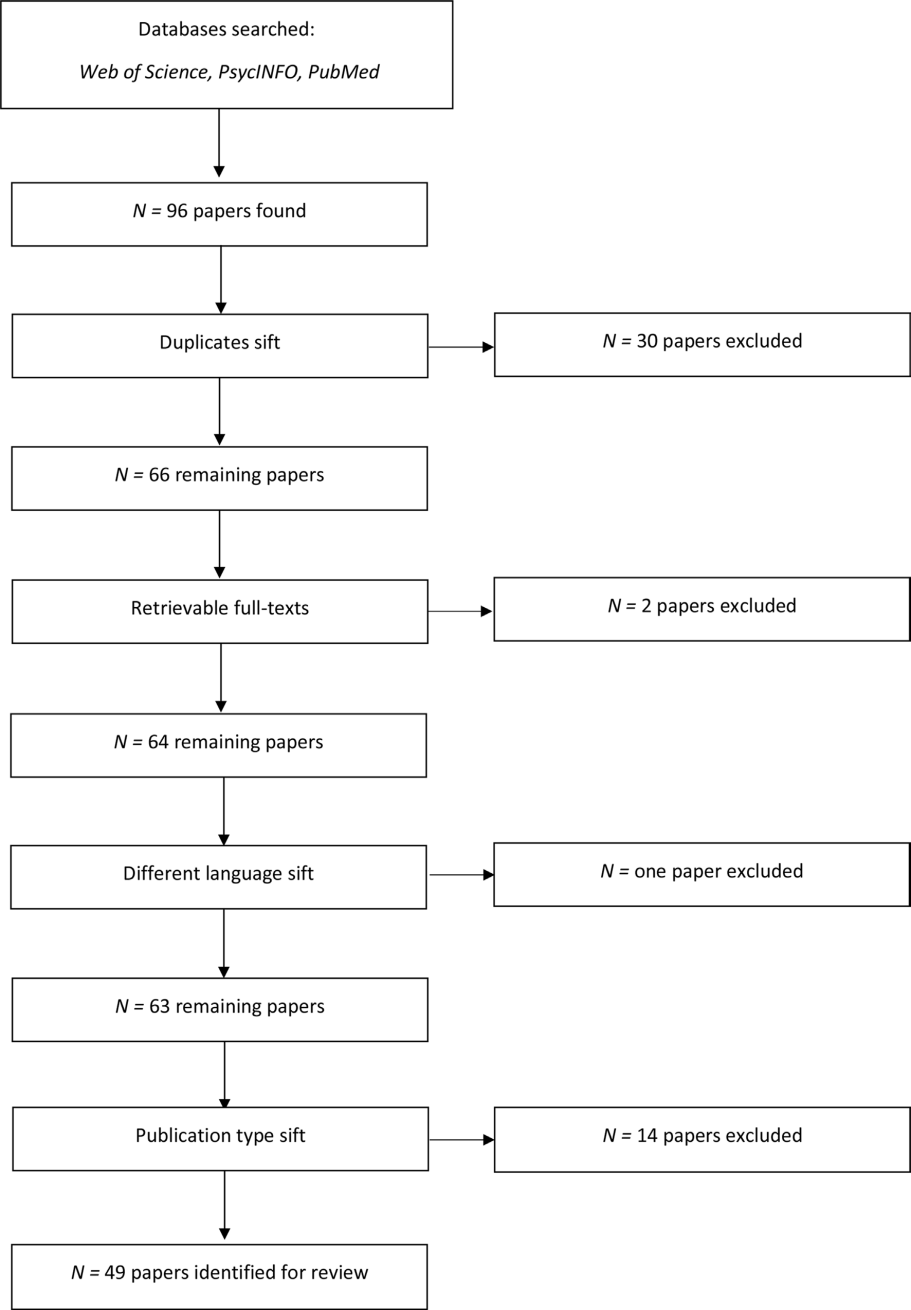
Flow chart displaying the search process.

Following title and abstract review, duplicate papers were removed. All other papers that appeared to meet the inclusion criteria were then assessed using the full text. At this point, any variations from the inclusion criteria were noted, and these papers were excluded. For instance, studies where the female subpopulation could not be distinguished from the male subpopulation were excluded. In addition to this, studies which were not published in peer-reviewed journals were excluded as indicated in the inclusion criteria (27–34). The search strategy is presented in **Figure 1**. All included studies were read, and key pieces of information were extracted including: sample size, recruitment process and participants, design of the study, aims, measures or tools used, main results, and the implications of the study. Thematic synthesis was then conducted.

## Results

Forty-nine studies were identified that met the inclusion criteria for this review (see **Figure 1**; see **Appendix A in the Supplementary Material**). As this study was the first of this nature concerning female gaming, the included studies represented research using various methodologies, such as clinical trials, experimental studies, and case studies, as well as other methodologies. Four main types of research were identified: i) the benefits of female gaming; ii) why women might play video games less than men; iii) perceptions and realities of female characters within video games; and iv) women’s position in gaming culture. The results section briefly outlines each of these. A few studies had material which could be included in more than one category.

### The Benefits of Female Gaming [*n* = 11]

Within the included studies, several considered how women engaging with video games might have a beneficial outcome. These comprised research examining clinical or environmental interventions (35–40), investigating cognitive and social learning, as well as strategies developed through game play (36, 37, 41–45). Studies that investigated the utility of video games to enrich an aspect of the participants’ life were proportionally more recent than those which considered other features of female gaming (35–40). Clinical interventions considered the physical (36, 37, 39) and mental benefits (35, 37, 38) which could be offered to women who interact with video games. All of these studies saw improvements in their participants’ abilities or health. However, they had relatively small sample sizes (ranging from two seniors (37, 40) to 23 women (37). The majority of studies also recruited from only one website (35, 38, 40).

Despite these limitations, video games were shown to have physical benefits, such as encouraging exercise in adults with lower mobility due to age and illnesses (37, 38, 40) and relieving pain symptoms in participants with fibromyalgia syndrome by offering cognitive distraction (38). Female gaming was also shown to improve mental well-being because video games were demonstrated to be acceptable psychotherapeutic tools to assist mental health recovery in adolescents (35). Gaming positively influenced executive functioning ability in women with urinary incontinence (37) and attention in elderly individuals (40). Two studies considered how gaming environments might be utilized as a teaching tool within undergraduate student populations (36). In both studies, it was observed that learners with less gaming experience showed lower levels of presence within the environment than others and that women were just as likely to succeed with this type of educational intervention as men (36, 39). However, DeNoyelles and colleagues (36) concluded that college-age women were less likely to be gamers. Therefore, they suggested that female non-gamers might struggle slightly more due to less gaming experience and suggested standardized support according to gender.

Even at an early age, it has been demonstrated that girls have similar abilities to gain strategy and performance skills by playing video games compared to boys (41). This was shown in 104 children (second to fifth-grade) where frequency of gaming was viewed as a better predictor of gaming performance and strategy than gender (41). This was reinforced by Olson and colleagues (45) who considered the main differences between video game play of males and females to be the amount of time spent playing and the types of video games. In a sample of 1,254 children taken from two schools’ seventh and eighth grade classes, it was shown that gaming was very common with 93.6% of children playing video games over the past 6 months but that boys were much more likely to play over 15 h a week in comparison to girls (45).

In a cross-country study of 145 young girls (*M* = 11.3 years) who played video games online, it was shown that prosocial gaming exposure to typical online video games (e.g., MMORPGs) had a strong relationship with perspective taking and sympathy, suggesting less severe violence acceptance (43). Within in-person co-playing, a subsample of girls demonstrated heightened prosocial behavior and stronger emotional connection when engaging with their parents (44). Again, this is evidence that video game playing has a positive impact on how girls develop their cognitive abilities, including social interaction. Alternatively, Olson and colleagues (45) demonstrated that more boys played video games than girls and that gaming could be utilized as a male anger management strategy. This could be reflective of how adolescents are primed to cope with emotional responses.

It has also been found that adult female gamers need less input when evoking response reactions than female non-gamers and have a greater neural plasticity which enhances this ability due to the familiarity of movements which are needed in gaming (42). This was verified in two studies by Gorbet and Sergio (42), in which they showed that playing video games has beneficial consequences on visuomotor performance but that these brain patterns are different from previous studies observing male responses, which may indicate different ways in which male and female brains react to problem solving within video games. Nonetheless, video games have a positive impact on muscle movements and response times to stimuli, as well as enhancing brain plasticity (42).

### Why Women Might Play Less Video Games than Men [*n* = 17]

Traditionally, women are thought to play less video games than men (13, 14). Several studies included in this narrative review discussed this consideration and offered reasons why female gaming is less common. Notable was the influence of gender expectations among those that engaged with playing video games. As previously mentioned, Olson and colleagues (45) attributed gender differences in time spent gaming to how social gender identities influence how children play, although male and female children play for the same reasons (e.g., fun, creative engagement, and emotional coping). One study found that if women play as often as men, they tend to reach similar levels of success within the game, discouraging the belief that women are less skilful at gaming (46). However, they also noted that female players tend to play less or stop earlier than their male counterparts, which they argued was due to gender expectations and peer community, e.g., game play was viewed as male dominated (46). These implications originated from two large studies of 9,483 players and 18,000 responses across two online video games. The vast majority of both populations were men, 82% and 74.5%, respectively (46); thus, from the respondents alone these studies indicate a community heavily featuring men.

The lower levels of female gaming may also be due to the nature of video games and the relationship with personal traits. For instance, violence and aggression are the focal point of many video games, such as FPS games. It has been suggested that repeated exposure to violent video games may elicit more aggression from boys than girls (47). A study of 98 adolescents from China indicated that there was no difference in reaction time to aggressive words when girls were primed by violent and non-violent video games, but that boys scored significantly higher when playing violent video games (47). This finding is supported by previous results (48) suggesting that violent-sexist video games are related to masculine beliefs (e.g., aggression and dominance), and therefore reduced empathic feelings when considering violence towards women. This might suggest that girls play video games less frequently than boys due to the level of aggression required by some video games. However, Ferguson and Donnellan (49) found contradictory results when running confirmatory analysis of Gabbiadini and colleagues’ (48) three-way interaction between game condition (neutral, violent, or violent-sexist), gender, and avatar identification. This demonstrated that there was no indication of a main effect of game content on empathy towards girls and that masculine beliefs were affected only slightly (*p* = 0.049). The authors suggested that this inconsistency of results would likely be reduced by a cultural research shift to preregistered studies and a focus on the validity of published results (49).

Generally, it is argued that exposure to violent video games can increase aggressive behavior and that this motivation can be higher when playing as a same-sex character (50). This second point relates less strongly to female gaming due to there being fewer female characters to act as (50) and because violence in video games causes women to disengage earlier (51). Within their study of 444 students across two universities, Hartmann and colleagues (51) suggested that women had limited exposure to violent video games due to trait empathy, which caused more anticipated guilt while playing, and therefore reducing enjoyment. Both men and women often select male avatars, which has been associated with more aggressive game play (52). For women, the selection of a male avatar may negatively influence the level of identification with the avatar. This may lead to women playing less often than men not due to aggression but due to less presence with characters within the video games. This conjecture is supported by studies from Eastin (53), which indicate that same-sex avatars encourage greater levels of presence within players, influencing aggression (54). Therefore, it is possible that female gamers do not succeed as much within violent video games as they often have to play an opposite-sex character competing against opposite-sex characters, reducing their presence and aggression, thus causing less success when leveling up or mastering the game.

Related to this, Norris (54) explored how individuals with aggressive personalities might interact with computer use and gaming in a population of 430 women recruited online. The population was split by those who were gamers and those who used chat rooms. Gamers were not found to be more aggressive than chatters, but more frequent game play was associated with more aggression within gamers (54). Using computers was thought to be masculine, with women who scored highly for masculine traits (e.g., being active, independent, and competitive) being shown to have higher levels of computer use (54). This provides some evidence for the association between game play and aggression. However, it does not consider the type of video games that the gamers in this sample played or the violent content of these. Again, encouraging computer use and viewing gaming as a cross-cultural activity (rather than masculine activity) was suggested (54).

Alternatively, Ferguson and Donnellan (49) suggest that female gamers tend to get stressed within gaming not because of the hostility or aggression of video games, but instead the annoyance of the game not being suited to them or what they would naturally select to play. This would suggest that women play less than men simply due to annoyance that the video games are not developed with female audiences in mind, as much as their male counterparts. A major reason why women tend to play less video games than men is the coping strategies that are required to handle harassment online (55–57). One benefit of anonymous online game play is that individuals may change their gender (e.g., changing their avatar from male to female or vice versa), allowing them to explore their gender identities (55). However, this can also have negative consequences. For example, Crow and Watts (55) found that some male teenagers changed their gender online to gain help from other players in the game or to help get rare items. This reinforced the stereotype that female players were less skilled and seeking preferential treatment, thus generally having to deal with more harassment. Women acting as male characters online was viewed as a valid strategy for handling harassment (55–58). Indeed, Martey and colleagues (58) found that while men were more likely to switch their avatar´s gender, they often did not seek to hide their offline gender, unlike women. Gender switching was considered to be more of a strategic selection within this sample (58), which might be related to the older age of the participants, averaging 29 years in comparison to the adolescents in Crowe and Watts’ sample (55).

In their qualitative study of strategies for online harassment, Cote (56) noted that strategies for managing gaming environments were often used such as playing offline, blocking players, and needing to prove oneself within the game. In informal conversations with nine online gamers, four of whom were women, coping strategies again favored anonymity of gender, non-verbal play, and banding with other women when dealing with sexual harassment or expectations from other players (59). Female gamers were considered inherently different, in that their legitimacy was put into question and they were asked to “prove” their gender (59). This hinders women from having the same gaming experience as men, and it impacts on their progression within video games (57, 59). Fox and Tang (57) found that across multiple countries, a common technique to avoid harassment was to reduce communication either completely or by masking their voices. By doing this, coordination abilities with teammates were reduced, causing women to level up more slowly than their male counterparts.

Motivations for game play may also be indicated as a reason why women tend to play less than men. One study found that women playing *Diablo III* tended to be motivated by their partners acting as a proxy player for the partner’s character when the male partner was too busy to play (60). This often ended with women deciding to buy and play the game themselves, often playing longer and spending more money than men (60). While this might not be a usual way to introduce others to gaming, the study provided evidence that encouraging female gaming had a positive effect on women wanting to play video games more often. This could relate to women feeling that playing video games influences their value as a significant other, which was demonstrated by Kasumovic and colleagues (61) within their studies. Sexual interest and mate value were positively related to violent video game exposure among women, and this was discussed as being due to women feeling like more attractive partners by having this shared interest (61). Therefore, a motivation to engage with gaming might be related to self-perceived sexual attractiveness.

Not only do video games have some utility to elicit motivation through self-perceived attraction, but Song and Fox (62) suggest that romantic video games can relate to these beliefs and thereby motivate romantically inclined individuals to participate in this activity. This study found that women with higher identification with their avatars had stronger parasocial beliefs (the perception of the figure to be known socially, rather than seen as a fictional character) about the romantic target within the game. These individuals also tended to have stronger beliefs about idealized romance (62). In this sense, the motivation to play is related to their desire of maintaining the relationship with the character within game, similar to studies which indicate shared gaming is a positive aspect of the partner relationship (60, 61).

### Perceptions and Realities of Female Characters within Video Games [*n* = 12]

Nearly one quarter of the included studies observed female characters within video games. These are broadly separated into studies considering the appearance of characters (63–67), how these characters were used in the video games (68–70), and how the characters themselves influence gamers’ beliefs (71–74). A number of these studies considered the physical characteristics of female characters through video game covers or game representation within gaming culture (63–65). It was often noted that women were less featured than men on game covers and that when they were featured, this was in a highly sexualized manner with exaggerated bodies, particularly regarding size of breasts and buttocks and slimness of waist (63, 65–69). Fisher (65), who considered characters from video game magazines, suggested that women were represented as sex objects rather than actual characters or avatars for gamers. These studies noted that positive portrayals were rare and weak within presentation, which may further discourage video game use among women (65). Indeed, this may detract from how women interact with gaming culture.

Across 368 characters, it was shown that female video game characters were smaller than a typical American and had unrealistic body proportions (67). Worryingly, Martins and colleagues (67) suggested that the highest degree of photorealism within characters were those taken from children’s video games. Unsurprisingly, video games intended for older audiences tend to feature the most sexualized characters, with fighting video games having the highest sexualization overall (66). Sexualization of women within video games was not shown to be associated with the success of video game sales across a 31-year period (66). Alternatively, within Spanish versions of console game covers, longitudinal comparative analysis suggested that there has been a decrease in the presence of violence and sexual objectification in female characters in more recent years (64). It is key to note here that across countries, depending on laws and policies in different countries, the covers of video games may vary and that the results of Burgess et al. (63), Fisher (65), and Martins and colleagues (67) are related specifically to US game releases. Near (70) stated that in their study of 399 video games purposively sampled from US sales, having a woman in the center or alone on the game cover negatively impacted sales.

As noted by Burgess and colleagues (63), male characters were almost 4 times more likely to be portrayed than female characters on console video game covers and were given significantly more game-relevant action. Of the utility given to women within video games, female characters are more likely to be secondary to the story of the game than males, and their role is typically sexualized. This was shown across 571 video games with playable female characters (66). This relationship is not demonstrated in the sample of 12 contemporary video games of 22 characters conducted by Jansz and Martis (69), who found that while male characters dominated video games, there was equal gender distribution between leading protagonists. Alternatively, female support characters were considered to hold more dominant positions compared to their male counterparts who were considered to be submissive (69). On the other hand, when looking at how children interpret physical features of characters, it was suggested that strength or masculine characteristics, such as athletic arms, are a translation of the character’s abilities rather than just for appearance (68). While this study was only conducted with 19 children, seven of whom were girls, it suggests that the appearance of a character is acceptable if it is representative of the abilities the characters hold.

However, while this is an interesting consideration of how children might interpret characters’ bodies, there have been several studies showing that a game character’s appearance may negatively impact how people perceive their own bodies (67, 71, 74). Martins and colleagues (67) considered that the high level of photorealism within children’s video games could influence body dissatisfaction at an early age and negatively impact how children perceive healthy bodies. For university students, the effect of characters’ bodies on self-perception has been demonstrated in both males and females, with both studies demonstrating reduced self-esteem (71). Interestingly, the female study evidenced that although self-esteem was impacted, body satisfaction was not, and this was attributed to female gamers considering the unrealistic comparisons being made (71). This might be a reflection of a smaller sample being used in the female study (32 participants compared to 51 within the first study) (71), or it could be a representation of video game priming at earlier ages.

Another study, which was more recently published, also examined male and female university students across two studies (74). They found that video games featuring hyper-idealized character bodies had a positive impact on body image satisfaction and attitudes among women but worsened these beliefs among men (74). These studies were conducted with a larger pool of students (149 female students and 197 males) (74). However, with regard to abilities, it was considered women might make downward comparisons based on the empowerment of physical capabilities by the avatar (74). In children, the physical traits representing particular abilities were viewed as positive, whereas in adulthood comparisons related to body may be more apparently negative.

One concern that is apparent from studies examining avatar bodies is the impact that they can have on the acceptability of violence towards and rape of women. Among 141 undergraduate students, it was found that following violence against women within video games, sexualized objectification and condoning rape myths increased in male participants (72). It was considered that the level of exposure and increased realism of the game influenced these attitudes so as to appear more acceptable (72). Again, for adolescents, playing as sexualized female characters indicated greater acceptance of rape myths and tolerance of sexual harassment (73). These studies indicate the influence of sexualized characters within video games and highlight how characters’ appearances can have negative influences on beliefs which may translate to real women and which could appear as sexual harassment to women offline. Alternatively, when designing female characters in video games, a sample of 14 females aged between 14 and 75 years demonstrated that they thought professionalism and interpersonal relationships were more important to a character than appearance, emphasizing behavior over physical characteristics (75). From this type of understanding, game designers might be able to develop avatars which encourage more women to play video games and convey less stereotypical roles.

### Women’s Position in Gaming Culture [*n* = 14]

Women are considered to be less engaged with gaming than men (12, 13, 76). It is possible that this extends to the culture as well as game play. However, despite this gender bias towards men, women are a part of gaming culture (23). This is emphasized by their positions as designers, gamers, and as users of gaming technology. Nevertheless, gaming culture is still considered to be a male-dominated environment (76), and the competence of women in these positions is often questioned (59, 77–79).

In a commentary paper, Harvey and Fisher (76) discussed the context of how women in gaming culture are perceived. Their study emphasized the challenges of being in this environment, particularly in the position of a video game designer (76). Within their commentary, they claimed that female designers received attention for physical appearance and as a “token female” rather than being considered for their abilities of game design. Arguably, this mimics the harassment seen within the online gaming environment itself, and because of these issues, it was noted that there was a constant problem of visibility as a female designer (76), again relating to the coping strategies observed to deal with online harassment (56, 57). However, it was also acknowledged that some of the hostility came from other women within gaming culture, promoting exclusivity, invalidating other players, and being unsupportive of other women within gaming (76). This was likely associated with women consistently having their position within the culture challenged.

A clear example of this discrimination is described by a study (77) which included the argument between Ryan Perez (a game journalist for the *Destructoid* video game blog) and Wil Wheaton (an American actor) regarding Felicia Day, a prominent female gamer. This incident included Perez slandering Day, reducing her to a sexual image, instead of an actual gamer. Perez suggested that she had poor gaming skills, and Wheaton defended Day (77). From this interaction, the bias against women in gaming culture is evident, considering that the attack from Perez was unprovoked by Day, her behaviors, or her fans. It is important to note that other men within gaming culture do not share these beliefs (including Wheaton), and this might encourage resistance against such comments in the future.

In the incident against Day, her competence as a gamer was questioned (77). This is a common pattern seen within gaming culture (79). Across two studies, Kaye et al. (79) demonstrated that avatar gender has an impact on how competent a player is considered to be. In their initial study, women with male avatars were considered to be more highly skilled than women playing as female characters, an effect that was not seen within male gamers. The second study attributes this to gender-role beliefs predicting sexist beliefs across MMO games and FPS games (79). Again, this relates to the gender swapping seen in previous studies as well as the responses which are attributed to this behavior (55). Within their small study, Linderoth and Ohrn (59) found that players were typically assumed to be male and therefore more competent, reinforcing the idea that women need more help or favors within video games.

Despite this study’s small sample size and low number of female gamers (four participants), it is evident that there is a bias in favor of men as gamers seen across research (59, 79, 80). However, this bias is not just among men. In their study of 39 female university students, Vermeulen and colleagues (80) demonstrated that women take gender as an indicator of skill when gaming, and they experienced more stress when playing against men. Considering this issue, the authors (80) suggested that this reaction was based on competitiveness as a perceived skill, where this competitive response was greater against women. The belief that female gamers are less competent when using technology is also seen in computerized assessments and by their own self-perceptions (78). This study demonstrated among a sample of 407 adolescents that boys had higher levels of computer game self-efficacy (78), most likely related to earlier priming of computerized technology when young (e.g., gaming at a younger age). Furthermore, the avatar’s appearance can impact self-efficacy in gaming. In a study of 328 university students, playing as sexualized female characters negatively impacted women’s self-efficacy when considered against non-sexualized characters (81). Students stated that this was based on considering the women’s capabilities, both cognitively and physically (81). This indicates that there are gendered beliefs towards women within video games when they are represented in a hyper-sexualized way, which may influence external considerations regarding women’s abilities.

Although gaming culture is mostly viewed as comprising men, a literature review evaluating 10 video games suggested that gamers were more evenly distributed in gender (23). As gender visibility is often limited as a coping mechanism within game play (57, 59), this study suggested that men are more visible, allowing them to more easily identify with the “gamer” label (23). However, it does appear that there are fewer women who play these video games more heavily due various game play elements such as violence, aggression, and/or representation, and this may explain why women stop gaming at earlier stages than men (46, 49, 53, 56, 57, 59). Paaßen and colleagues (23) go one step further and suggest that the stereotype of gamers is accepted into the identity of male gamers, whereas women are required to be either a woman or a gamer, marginalizing women within gaming culture.

## Discussion

The aim of this narrative literature review was to provide a comprehensive overview of empirical and theoretical studies concerning female gaming and the position of women within gaming culture from an individual and cultural perspective. The 49 studies in the review of female gaming were classified into four types of research studies, namely, i) benefits of female gaming, ii) why women play video games less than men, iii) perceptions and realities of female characters within video games, and iv) women’s position within contemporary gaming culture. Each of these will be discussed below.

The studies reviewed indicate that playing video games has a wide variety of benefits for women, in terms of both physical and mental health benefits. Empirical research suggests women have much to gain from interacting with video games at a variety of ages and by playing different types of video games (17–19). Gaming by females in childhood and adolescence may enhance cognitive, social, and/or movement abilities and enhance engagement with educational tools (18, 19). Indeed, the benefits of enhanced brain plasticity and reaction times may be advantages for offline interactions, such as sporting activities or problem solving. Integrating gaming more fluidly into female’s lives may improve mental and physical health long term, as well as reduce isolation through online communication (19).

This is in line with previous research (82) showing that video games have many beneficial effects on gamers (both genders, young and old) and are used in various contexts, including video games as physiotherapy and occupational therapy, distractors in the role of pain management, cognitive rehabilitation, improvement of social and communication skills among the learning disabled, alleviating symptoms of impulsivity/attention deficit disorders, therapeutic benefits in the elderly, psychotherapeutic settings, health care, treatment of anxiety disorders, and psychological well-being. Moreover, experimental research (83) indicates that playing video games can result in improved task-switching, better top-down attentional control (rather than bottom-up) and processing speed, and increased and quicker time perception. It is concluded that there has been considerable success when video games are specifically designed to address a specific problem or to teach a specific skill. However, generalizability outside the game-playing situation remains an important consideration.

A number of studies included in this narrative literature review were concerned with the question of why women appear to play less video games in comparison to men. Across the included studies which included both men and women, female participants were typically in the minority (46, 51, 55, 58), with the exception of the studies conducted by Yang et al. (52) and Lopez-Fernandez (17). It is possible that this is representative of the overall gamer population, as men appear to comprise a larger proportion of gaming culture, or inversely that gaming culture is catered for male gamers (22–25). Overall, the studies included here indicate that women are less encouraged to participate in playing video games due to negative expectations based on gender or experiences during game play, as well as video games being designed and developed in a way which is less enticing to women, including overly aggressive and sexualized content (84).

In addition to this, an important reason for why women tend to play less video games than men is the coping strategies that are required to handle harassment online, with women often playing male characters in order to avoid in-game harassment and bullying (15, 56, 57, 85). Kuss (85) also showed that males benefit from this strategy because they often play female characters in order to receive additional support from other gamers, suggesting that gender-swapping is a strategy that is applied by both male and female players and results in various benefits in terms of their game play and well-being, which was supported by another study (86), indicating that playing a female character in MMORPGs results in positive social attributes. However, Lopez-Fernandez et al. (19) have shown that female gamers do not swap their avatar gender to cope with the potential violence when gaming online. Moreover, females appear to look for different things in video games in comparison to men (e.g., relationship maintenance) (19, 18), and game designers should take this into consideration when developing video games for this growing audience. This, in itself, would impact how women are perceived within video games and that their abilities to gain high experience levels, rare items, and special capabilities are just as good as that of men.

The next major theme incorporated in the present review was the perceptions and realities of female gaming characters within video games. The results indicate that not only are female characters featured less frequently in video games, their representation often appears to be exaggerated and hyper-sexualized in terms of emphasizing their female attributes (i.e., size of breasts and buttocks, and slim waists), which may negatively impact on female gamers’ own body image given the representations of female bodies in video games do not correspond with the reality of female bodies (63). This is consistent with the scarce empirical research performed on female gamers at present (19). Upward social comparisons of oneself with others, as they frequently occur on social media sites, may in fact lead to feelings of decreased self-esteem and depression (18, 84), suggesting that this mechanism may apply similarly in comparisons between one’s female avatar and oneself, leading to decreases in well-being and symptoms of mental disorders, such as mood disorders.

Research has showed that having female characters prominently represented on video game box art decreases sales rates (70). Overly sexualized female characters in game can have a negative impact on self-perceptions and beliefs which may impact interactions and perceptions outside of the game. For instance, some video games propagate acceptability of violence towards and rape of women, increasing acceptance of the rape myth (72). Young and Whitty (87) explored why taboos, including rape, are violated in video games, and point out that the freedoms afforded by video games may negatively impact on gamers and their real-life interactions. Gamers can develop strong attachments to their online representations in the form of their avatars, and violence against them is distressing (88). Hypersexualized female bodies and condoning violence against female characters in-game may have negative impacts on gamers’ perceptions of themselves, others, and their interactions with females, suggesting game designers should carefully consider the inclusion of females with exaggerated female attributes and violence when developing video games (89).

Within younger audiences, it might be appropriate to have bodies which are representative of the special abilities held by the character, but ultimately from the sample of studies included in this review, it appears that hyper-exaggerated bodies can have negative influences on body satisfaction with women and to some degree with men. By considering this, game developers should be encouraged to indicate abilities through the character’s body, but in a less hyper-idealized manner because this may improve the gamer’s perceived body image. Indeed, this would allow young audiences to consider the behavior of the character to be integrated with their appearance and suit older audiences who are more concerned about the behavior and skills of an avatar. Furthermore, reducing the sexualization of females would have liberating effects in terms of how women are considered both within and outside of video games. Similarly, other socio-demographic features (e.g., ethnic, cultural, religious, or sexual) in the avatars shown could offer a bigger range of identities and tastes which could facilitate avatar identification without body image dissatisfaction and other problems which are currently causing the heteronormative video game content in gamers (89–91).

The final main theme covered in the reviewed studies was women’s position in gaming culture. Statistics suggest female gamers are increasing in number (1, 2), with female gamers significantly outnumbering males when it comes to mobile gaming (92), typically comprising “casual” gaming. Despite this, gaming culture still appears to be male-dominated (67, 76), while female gamers’ abilities as competent game players are put in question and not considered yet as “true” or “hard-core” gamers (24, 77). The experiences of women in gaming culture are mirrored within other fields with technology discrimination [e.g., electroacoustic composition (93)], otherwise known as “Programmed Inequality” (94) (i.e., despite the growing number of females, there are still barriers to entering and working in male-dominated environments; e.g., there appears to be a systematic neglect of technical training due to gender). This highlights that the problem of women having a valued presence in technological culture and industry is not new. Furthermore, it appears that there are no regional or time variations regarding this issue.

This review expanded on previous research and targeted specific outcome studies covering the topic of female gamers, but it is not without its limitations. One major limitation is that while the authors followed rigorous search methods to identify relevant papers, the review was limited to those published reports that the authors were able to locate. It is possible there are additional studies that cover this topic but were not included in this review (i.e., because of publication bias). The large number of outcome studies is in itself a strength in formulating conclusions that can be extracted about female gamers and their position in gaming behavior and gaming culture. Even for a scarcely researched topic such as this, the research team found a considerable number of peer-reviewed papers. However, there are also limitations, such as excluding papers in Asian languages given the large gaming culture in Asian countries, including China and Japan, many of which have developed sophisticated targeted approaches in preventing gaming addiction (95), mainly because it is considered much more of a public health concern in these countries than elsewhere.

Additionally, the specific scientific databases selected and the inclusion criteria used to conduct this review may have excluded some sources, especially from other disciplines outside of psychology and medicine, although *WoS* is an interdisciplinary database. Furthermore, expanding the review to female gaming from female and male perspectives could in some way have limited the views of this specific gender, although alarming findings have also emerged (e.g., technology discrimination). Finally, the present study is probably affected by generalizability bias. For instance, in terms of geographic location, out of the 49 studies that provided information, 45 were essentially located in the Western culture [i.e., 33 in America (27 in the United States, 4 in Canada, and 2 in Chile) and 12 in Europe (the Netherlands, Spain, United Kingdom, Italy, Sweden, Denmark, Germany, and Belgium); see Appendix A for details about the authors, location, and other methodological elements of the samples in each paper]. Thus, it is not possible to draw conclusions about the extent to which sample demographics across the studies in this review reflect female participants only, the population within a particular geographic region, or across the nation. The findings only reflect those based on gaming research in Western culture.

Overall, the included studies reflect the difficulties that women experience within video games among the general community of gaming. Women are still harassed, belittled, and considered less able than men when it comes to gaming, by both men and women. To hold an identity within gaming culture, women must follow strict rules about how they conduct themselves and hold views which emphasize that they are part of gaming culture and that other women are not part of gaming culture. Building on from this, women need to support each other openly and visibly in the community, with reinforcement from men. The solidarity campaign HeForShe (https://www.heforshe.org/en) started by the United Nations is an excellent case in point addressing gender equality, whereby both women and men are encouraged to commit to standing in “solidarity with women to create a bold, visible and united force for a gender equal world.” In the context of women’s position in gaming culture, this contemporary feminist movement helps support the view of women as being just as capable and skilful gamers as men and require just as much respect and recognition from gaming culture, which they are part of. It may influence beliefs that women are inferior within gaming and encourage more females to play video games. It may also open up communication in such a way that harassment is reduced, and designers consider video games with women as their audience more so than they have done previously. According to the ESA (96), 45% of United States gamers are women and are therefore a very large market that can be targeted by the gaming industry.

Taken together, the research cited in the present narrative literature review suggests female gamers are a growing population. Gaming appears to offer a variety of benefits for them, from cognitive and psychological benefits to physical and social benefits. However, women still face an over-sexualized representation of female in-game characters, online harassment, and an expectation that they are less skilful players in comparison to male gamers. Furthermore, contemporary video games do not sufficiently target female gaming motivations and gaming-related interests, despite the number of female gamers increasing. Based on the outcomes of this narrative literature review, it can be suggested that the gaming industry should pay more attention to the needs and interests of female gamers given they are an audience to be taken seriously and now large in number. Moreover, research should be encouraged to specifically investigate female gamers’ motivations, as well as the psychosocial impact that in-game violence and hypersexualization of female characters has on their mental health and well-being, as well as their overall gaming experience. Longitudinal, qualitative, and psychometric approaches should be combined to offer a more comprehensive and holistic picture of the female gamer, including their socio-demographics, interests, and psychosocial environment of gaming, including the gaming culture they are part of.

## Author Contributions

OL-F, DK, and MG contributed to the conceptualization. OL-F and AW contributed to the data curation. AW conducted the formal analysis. OL-F, DK, and MG contributed to the funding acquisition. OL-F, DK, and AW conducted the investigation. OL-F, DK, and AW contributed to the methodology. OL-F conducted the project administration. OL-F, DK, and MG contributed to the resources. OL-F conducted the supervision. Writing of the original draft was done by OL-F (Abstract and Introduction), AW (Method and Results), and DK (Discussion). Review and editing were done by OL-F, DK, AW, and MG.

## Funding

The present study was supported by the Psychology Department QR Funding at Nottingham Trent University, through a Kickstarter grant (2018) awarded to OL-F to develop studies on “Female gaming: A cross-cultural study of pathological and non-pathological gaming *via* multi-platforms.”

## Conflict of Interest Statement

The authors declare that the research was conducted in the absence of any commercial of financial relationships that could be construed as a potential conflict of interest.
