# Supplementary material for: Female Gaming, Gaming Addiction, and the Role of Women Within Gaming Culture: A Narrative Literature Review
Source: Front Psychiatry. 2019 Jul 10;10:454. doi: 10.3389/fpsyt.2019.00454 (PMC6635696; doi:10.3389/fpsyt.2019.00454)
Supplement: Supplementary file 1 [file Table_1.docx]

| Authors | Sample | Design | Aim | Measures | Results | Implication |
| --- | --- | --- | --- | --- | --- | --- |
| Anderson & Murphy, 2003  U.S.A. | 91 female undergraduates in U.S. (*M* = NR, *SD* = NR). Recruited from introductory psychology class for course credit.  VVGS with female protagonist, *N =* 33.  VVGS with male protagonist, *N =* 30.  Control non-violent game, *N* = 27. | Empirical : Survey -  Cross-sectional design using reaction times to video games and self-report questionnaire. | To conduct a replication of VVGS effects on aggression in young women and to provide a preliminary look at the potential moderating variable of gender of controlled character on VVGS effects. | Modified version of the TCRT: assesses aggressive behaviour (Anderson et al., 1999; Carlson et al., 1989; Giancola & Chermack, 1998)  Questionnaire focused on motivation (Bartholow & Anderson, 2002) | Brief exposure to VVGS increased aggressive behaviour. VVGS effect on aggression was not mediated by instrumental aggressive motivation, but partially mediated by revenge motivation. Motivation may be greater when game player controls same-sex game character. | Results support hypothesis that brief exposure to VVGS can increase short-term aggressive behaviour by young women, possibly stronger when same-sex game character. With a larger sample this needs to be explored to understand whether effects are maintained. Relates to evaluation of male aggression with regard to VVGS – shows effect can be found in females too. Further research to identify features of VVGS which may impact aggression, it is possible that these will have long-term effects of repeated exposure. |
| Barlett & Harris, 2008  U.S.A. | Study 1:  51 males (*M =* 19.2yr, *SD =* 4.6yr)  Recruited from General Psychology class for course credit in a U.S. Midwestern University.  Muscular condition, *N =* 27.  Non-muscular condition, *N =* 24.  Study 2:  32 females (*M =* 18.9yr, *SD =* 1.1yr).  Recruited from freshman undergraduate students in a U.S. Midwestern University. | Empirical : Survey -  Cross-sectional design using video games and self-report questionnaire. | Study 1:  Determine if playing a video game emphasizing muscular male bodies would increase negative body-images in males.  Study 2:  Determine if playing a video game that emphasized the thin female body would increase negative body-images in females | Body Esteem Scale: assesses state body esteem (Franzoi & Shields, 1984)  Demographic questionnaire: age, school year, ethnicity, height, weight  Suspiciousness questionnaire: assess whether participants could infer predictions of study  Study 1:  Swansea Muscularity Attitudes Questionnaire: assess attitudes toward muscularity (Edwards & Launder, 2000)  Male Body Image and Esteem Scale: measures body esteem in men (Barlett et al., 2005; Markunas et al., 2003)  Study 2:  Body Shape Questionnaire: measured body satisfaction, the cognitive component of a negative body-image. (Cooper et al., 1987) | Study 1:  Results suggested that when male participants played a video game that emphasised muscular male bodies, they had an increase in their negative body-image. Significant decrease in drive for muscularity after playing video game for those in non-muscular condition.  Study 2:  Women participants felt significantly worse about their bodies after playing video game. Marginal decrease in positive feelings of their sexual attractiveness. Body self-esteem affected but not body satisfaction. | Mass media format of video games can contribute to negative body-images in both males and body self-esteem in females. Possibly that decreased drive for muscularity in males and body satisfaction not significantly impacted due to unrealistic comparison of game characters. In particular, for some female gamers this might result in up-wards comparison in females own body favour. Need to determine which media format is most impactful. |
| Beck, Boys, Rose, & Beck, 2012  U.S.A. | 141 undergraduate students, 61% female, (*M =* 21.6yrs, *SD =* NR).  Recruited from two U.S. universities in the Northern Midwest and the Southern Midwestern.  (group allocation NR) | Empirical : Experiment -  Solomon four-group designs | To explore the influence of the negative, sexist portrayal of women and violence against women in video games on rape-supportive attitudes. | Illinois Rape Myth Acceptance Scale: assesses rape myth acceptance (Payne et al., 1999)  Video game survey: measures exposure to video game violence (Anderson, 2000) | Findings indicated that sexual objectification of women and violence against women in video games do increase rape myths in male participants. Degree of exposure not supported in increasing negative attitudes toward women. | Increased realism in video gaming may escalate the influence of gaming on attitudes and behaviour; particularly violence and objectification of women. Indicates that increasingly realistic sexually aggressive violence can influence men’s attitudes towards women and acceptability of rape. |
| Behm-Morawitz & Mastro, 2009  U.S.A. | 328 undergraduate students from a U.S. Southwestern university, 63% female (*M = NR*, *SD = NR*).  Voluntary recruitment. | Empirical : Survey -  Cross-sectional design using video games and online questionnaire | To investigate the short term effects of exposure to sexualised female video game characters on gender stereotyping and female self-concept in emerging adults. | PQ: measures presence and items related to character identification (Kim & Biocca, 1997; Witmer & Singer, 1998)  Rosenberg’s Self-Esteem Scale (Rosenberg, 1965): assess self-esteem.  General Self-Efficacy Scale (Sherer et al., 1982): assesses self-efficacy.  AWS: gender attitudes and beliefs (Spence & Helmreich, 1972) | Playing the sexualised heroine resulted in lower self-efficacy in comparison to playing the non-sexualised heroine or no video game at all. Less favourable judgements about women’s cognitive and physical capabilities. The level of presence experienced had no significant effect on these relationships. | Cautiously suggest that playing a sexualised video game heroine unfavourably influenced people’s beliefs about women in the real world. Appearance of women impacts the consideration of her abilities within a given situation. |
| Bergey, Ketelhut, Liang, Natarajan & Karakus, 2015  CANADA | 407 middle-school students from sixth, seventh and eighth grades, 51% female (*M/SD =* NR).  Recruited from 9 schools by science teachers (location NR). | Empirical : Survey -  Computer based task followed by electronic questionnaire in preparation for computerised assessment | Examine how achievement on a science assessment that requires scientific inquiry skills in IVE relate to prior and subsequent scientific inquiry self-efficacy beliefs. Does low computer game self-efficacy impact science assessments that take place within IVE interactions. | SETS self-efficacy: scientific inquiry into self-efficacy (Ketelhut, 2011)  SETS computer game self-efficacy (Ketelhut, 2011): assesses self-efficacy for computer gaming ability | Results from path analyses indicated that prior scientific inquiry self-efficacy predicted achievement on end-of module questions, which in turn predicted change in scientific inquiry self-efficacy. Computer game self-efficacy was neither predictive of nor predicted by performance on the science assessment. Boys had higher computer game self-efficacy compared to girls; multi-group analyses suggested only minor gender differences in how efficacy beliefs related to performance. | Gender difference in self-efficacy beliefs may shape willingness to engage or persist with computer game-like IVEs. Researchers should measure computer game self-efficacy to evaluate whether beliefs influence engagement with IVEs. Despite gender differences shouldn’t rule out use of IVEs for educational uses, and offer further feedback or support for those with lower self-efficacy beliefs. |
| Blumberg & Sokol, 2004  U.S.A. | 104 children; 46 second- grade (*M =* 7.4yrs, *SD =* NR) and 58 fifth-grade (*M =* 10.5yrs, *SD =* NR), 41.3% female.  Recruited from multiple schools across New York. | Empirical : Qualitative study -  Brief structured interview followed by observed video game task | Examine potential differences in cognitive strategy between girls and boys. | Dependent variables: highest level completed, highest level attained, character lives, lives lost, number of games started.  Strategy observations: assesses internal/external strategies. Internal: learning to play by reading instruments/trial and error. External: asking for instructions or watching others’ play.  Interview questions: frequency of play, understanding of how to play, previous play of game | Frequent players had significantly better game performance than infrequent players. No significant differences of internally based strategy between gender. Greater proportion of internal strategy within frequent players and by older children. | Gender not implicated regarding internal or external game strategy or performance. Informal learning time spent by children playing video games, and differences between gender and game use may have differing ramifications for cognitive gains. |
| Burgess, Stermer, & Burgess, 2007  U.S.A. | 225 console video game covers.  Sampled by genre of game then cross-referenced for overlap, and checked against sale rankings on Amazon to ensure inclusion of 50 top selling video games per console; X-Box, PS, Nitendo Gamecube. | Empirical : Qualitative study -  Content analysis of console video game covers | Whether male characters are portrayed more often and if female characters are more hyper-sexualised than male characters. | Operational definitions: type of character, concepts for anatomy, concepts for violence, objectification, male body type, female body type | Male characters were almost four times more frequently portrayed  than female characters and were given significantly more game relevant action. However, in spite of their less frequent appearance, female characters were more likely to be portrayed with exaggerated bodies, and often objectified - sexiness. | Limited portrayals of women could suggest that women are less capable or less influential than men. Negative portrayals of women could make female gamers think they should look a particular way. Physical attributes in many games not naturally attainable. Possible ramifications on self-image when comparing to these characters. |
| Carrasco, 2016  CHILE | 5 psychotherapists recruited from two Chilean public health institutions and private practice. 15 adolescent females with mild to moderate depression (12-18yr, *M/SD =* NR) recruited by their psychotherapist (*M =* NR, *SD =* NR) | Empirical : Experimental study  –  Intervention with interviews | Test and evaluate use and acceptability of video game as psychotherapeutic tool for depressed adolescents. | Acceptability scale: expression of opinions about value and benefit from playing game | Some patients valued the video game in terms of learning helpful social and mental health-related behaviours. Therapists manifested that a video game could be a useful complementary tool for psychotherapy of adolescent girls. | Future developments of video games for mental health are worthwhile as majority of patients found this to be acceptable. This study provides some idea on how to incorporate psychotherapeutic notions in ludic environments, which may help initial progress with social aspects of recovery in mental health. |
| Castillo & Doral, 2016  SPAIN | 203 female characters across 134 console game covers taken bought in Spain between Oct2011-Oct2015. | Empirical : Qualitative study –  Content analysis of console video game covers | To investigate the evolution of women’s image on video game covers. | Variables considered: number of female characters, number on the cover, role of character, company of character, level of nudity, beauty, age, sales presentation, involvement, presence of violence, console system, year of commercialisation, PEGI, genre of game, sensuality, art style, infantilisation, level of prominence | Longitudinal comparative analysis showed decrease in presence of violence and sexual objectification. Observed association between female characters and other variables (e.g. violence, infantilisation) that can distort conception of sexuality. | Video games increasingly important socialising tools within children and adolescents – important to understand how transmitting sexist stereotypes may lead to negative behaviour against women. Reduced objectification of female characters may encourage female game play, although if male characters are still often used in violent settings, but females are not this could implicate inability of females. |
| Chou, 2011  TAIWAN | 14 females (age 14-75yr, *M/SD =* NR)  Recruitment discussed. | Empirical :  Experimental study  -  Cross-sectional design using in-depth interview to form matrix | To investigates nature of the female role model and analyses the relationship matrix for designing female characters in game design, using UCD | FQD: compares female character descriptions rated on importance and design requirements from two-experienced character designers.  QFD: management framework and systematic process for motivating business from customer needs. (Cohen, 1995; Zhao et al., 2005) | Findings showed core values regarding professionalism and interpersonal relationships were more important than appearance. Matrix based on interviews suggests that during development stage, behaviour was more important than appearance. | Suggest collaborative use of user-centre design and participate design. This would represent female subjects desires and proposals for in-game changes, which designers can use to develop characters combining information from users and own findings in the field. This could encourage games to be more appealing to women and represent less stereotyped characters. |
| Cortes-Picazo, 2016  CHILE | *N* = 19; 7 females (children in final years of primary school; *M/SD =* NR). Recruited from four schools in Talca. | Empirical: Research-action through a qualitative approach (observation and interviews). | To identify and analyse the dominant discourse around the transgression  of traditional identities of women in video games. | The main task for the children was to draw their own character for a video game, after viewing a demo about a video game with a female main character (*Ayumi*, from the ‘Blade of Time’, 2012; *Lara Croft* in Tomb Raider, etc.).  Draws: documental evidence provided by the children.  Photographic data: of these drawings.  Audio-visual recordings and transcriptions.  Interviews with children. Some questions were: What is your video game about? What is the role of your main character? And what are the characteristics of your main character? | Children provide their feminine main characters with masculine characteristics; for example, they translate violence, the use of arms, or actions that could promote the death of one rival in their female characters, e.g., a warrior princess with special powers to fight against mutant dogs.  Children use a Japanese aesthetic, such as oriental faces, hair, clothes like in manga cartoons. Girls usually use a black pencil for their character’s black hair, but they do not masculinize the feminine figures as masculine. This is only done by boys, as well as in the industry. Finally, children usually provide a narrative structure to their tragic fictional video game stories, where the female character should assume roles and life adventures to defend the ones who have suffered unfair life experiences. | From a children’s perspective, female characters in video games usually have strong physical characteristics to be able to fight. It seems women are migrating to traditionally male spaces (video games) from a techno-feminism perspective. The first step has been modifying the aesthetics of these female roles to develop themselves using masculine criteria in video games. |
| Cote, 2017  U.S.A. | 37 female (all self-identified gamers) (*M =* ~25yr, *SD =* NR).  Recruited as part of a larger humanistic study regarding games and gender; based in multiple countries with majority coming from the U.S.A. | Empirical :  Qualitative study  -  Gender-based case study using grounded theory | To explore women’s strategies for coping with online game-related harassment | Semi-structured interview: topics of discussion NR | It was shown that women are an active audience within game culture, carefully managing their gaming environment to help ensure positive experiences, e.g. playing offline or with known players. Camouflaging gender, developing skills and own strategies were found to be more useful than gaining help from male counterparts, blocking harassers or being flirtatious. | Female players were capable of coping with harassment but had to adjust behaviour because of harassment. Overall culture change for gaming would be more effective to include more diverse players or character representations. This may change harassment patterns over time and coping strategies. |
| Coyne, Padilla-Walker, Stockdale & Day, 2011  U.S.A. | 287 adolescents (*M =* 13.3yr, *SD =* 1.1yr) from 106 single parent and 190 two-parent families. 35% female adolescents.  Recruited from the Flourishing Families Project, large U.S.A. Northwestern city. | Empirical :  Experiment study  -  Cross-sectional design using video task, interview and questionnaires | To assess the relationship between parental co-play of video games and behavioural and family outcomes | Internalizing and delinquency (Nikken & Jansz, 2006; Nikken et al., 2007): assesses depression/anxiety, and delinquency.  Aggression: self-restraint dimension of aggressive behaviour measure (Weinberger et al., 1979)  Modified version of the Kindness and Generosity: subscale of Values in Action Inventory of Strengths (Peterson & Seligman, 2004)  Parenting Styles and Dimensions Questionnaire-Short Version: parent connection to adolescent using warmth and connection subscale (Robinson et al., 2001)  Co-playing: technology or media in connection with parent | Time spent playing video games associated with several negative outcomes – heightened internalising and aggressive behaviour, lower prosocial behaviour. Co-playing video games with parents was associated with decreased levels of internalising and aggressive behaviour. In females, this also heightened prosocial behaviour. | Co-playing with adolescent females may be one way to stay involved with adolescent activities and negate negative effects of games; females and their parents reported stronger connection with each other, particularly when playing age-appropriate games. |
| Crowe & Watts, 2014  U.K. | 2161 online adolescent members, estimate 20% female.  (*M/SD =* NR).  Data taken from within Runescape in-world public and private chat mechanism, online interviews via Microsoft Messenger or in-game chat, and related forums and newsgroup postings. | Empirical :  Qualitative study  -  Longitudinal ethnographic study of virtual worlds | To explore adolescent practices in virtual spaces of online gaming communities | 1628 separate in-game observation sessions (4500 hours)  3247 in-game virtual interviews  50 forum threads  140 extended peer-to-peer discussions  20 material focus group interviews  23 game observations in material world | Gendered characters have different utilities, acting as a woman allowed players to gain help more successfully and feel “sexy”; however, they tended to be harassed and skills were less considered than when playing as a man. The ability to change sex can allow adolescents to explore sexual and social identities more easily. | `Virtual spaces allow adolescent to select their gender, however, their understanding of what it actually means to be that gender will be shaped by cultural experiences. The use of stylised representations creates issues as they glamorise and objectify aspects of gender. Virtual adjustable identify allows physical identifiers to be absent for a time, this may have negative or positive implications for the individual depending on their self-liking of their real-world identity. |
| deNoyelles & Seo, 2010  U.S.A. | 21 undergraduate students (13 self-identify as female, and only 2 consider themselves to be gamers).  18-24yrs. (*M/SD* *=* NR)  Enrolled in Communications and Technology course at a large U.S.A. Midwestern university, which utilised lab-based weekly Second Life sessions. | Empirical :  Qualitative study  -  Observational study of Second Life using blogs, “real world” observation of interactions with Second Life and 4 interviews. | To investigate how college students project their virtual identities and engaged with interaction in Second Life, and how this influences their learning of course content | Demographic survey: age, gender, previous experience with multi-user games and Second Life.  Second Life related blog entries: specific prompt for reflection, identity, exploration, environmental communication theories.  Interviews: description of class, Second Life, personal affiliation with gaming, reflection on activities in lab, avatar interactions and technical difficulties. | Analysis of multiple data sources revealed that conceptions of identity, beliefs of the nature of  the virtual world, and technical skill were primary factors which affected group cohesion and learning within the community. Particularly relevant with female non-gamers feeling that they were not translating personality or feeling able to engage. | Multi-user virtual environments can act as useful resources however; female non-gamers may see less benefit than male gamers. Possible that more support would be required for females within this IVE to encourage engagement with activity. However, this suggestion could have negative implications for self-efficacy of ability to learn in IVE if standardised support by gender was offered. |
| Driesmans, Vandenbosch & Eggermont, 2014  THE NETHERLANDS | 57 adolescents (*M =* 13.3yr, *SD =* .6), 22 females.  Recruited as a convenience sample during visit to a Belgian secondary school.  Female sexualised character, *N* = 29. (Sexualised character distinguished by her tight, revealing clothes emphasising slim body).  Nonsexualised character, *N =* 28.  (Nonsexualised character wore loose-fitting clothing, covering body and hair over the face to make it unclear if they were male or female). | Empirical :  Experimental Study  -  2x2 factorial design experiment | To investigate the effect of playing a video game with a sexualised female character on adolescents’ acceptance of rape myths and tolerance for sexual harassment | Rape Myth Scale: measures acceptance of false beliefs that deny and justify male sexual aggression. (Lonsway & Fitzgerald, 1995)  Tolerance for Sexual Harassment Inventory: assesses tolerance for sexual harassment(Lott et al., 1982) | Analyses of variance showed greater acceptance of rape myths and greater tolerance of sexual harassment in adolescents who played with the sexualized character compared to adolescents in the control condition. They did not find significant differences between boys and girls or any interaction effect between gender and game character. | Suggest that sexualised women in gameplay may increase adolescents’ acceptance of rape myths and tolerance for sexual harassment. Highlight attention to the use of these characters in videogames targeting adolescents. Concern that this may influence behaviours towards women. |
| Eastin, 2006  U.S.A. | Study 1:  76 female students (*M =* 21.0yr, *SD =* 2.5yr).  Study 2:  75 female students (*M =* 20.1yr*, SD =* 1.6yr).  Study 3:  81 female students (*M =* 21.7yr, *SD =* 2.0yr).  All samples: Recruited from large introductory communication class at Ohio State University.  (Group allocation: NR) | Empirical :  Experimental study  -  2x2 experimental design | Study 1:  To explore whether gender matching of participant and virtual character heightens levels of presence and aggressive thoughts experienced  Study 2:  Investigating the influence that human and CPU competitive gaming has on presence and aggressive thoughts as well as replicating results in study 1  Study 3:  Investigating whether opponent gender influences levels of presence and aggressive thoughts | PQ: consists of control, sensory, realism and distraction (Witmer & Singer, 1998)  Word completion task: assess aggressive cognition following brief exposure to violent content (Anderson, Carnagey, & Eubanks, 2003; Anderson et al., 2004) | Study 1:  Same-sex character representation did increase the level of presence experienced compared to opposite-sex representation but not significantly. Aggressive thoughts significantly differed by avatar gender, eliciting more aggression when same-sex than opposite-sex.  Study 2:  Data indicated that players with same-sex avatars experienced greater levels of presence than those viewing opposite-sex avatars. Aggressive thoughts significantly differed by gender representation. Same-sex avatar increased aggressive thoughts and did significantly differ between computer and human opponents.  Study 3:  There was not a statistical significance of influence between human and CPU opponents for presence, nor did the opponent gender when matching player gender. However, there was support between opponent type and aggressive thoughts, human opponents elicited greater aggressive thoughts, and opponent gender was significant. Male opponents produced greater aggressive thoughts than female. | The representation of character gender influences game experience, causing more aggression against same-sex avatars, particularly when CPU. These experiments indicate that female game players attend to self, opponent, and opponent type during game play. Possibly female gamers may not succeed as much in VVGs as often playing against opposite-sex characters, thus do not level up or master the game as quickly due to lower aggression. The ability of games to manipulate the characteristics of avatars and agents could produce new opportunities for gaming researchers to understand perceptions of self, group, and others in both competitive and cooperative situations. |
| Ferguson, Trigani, Pilato, Miller, Foley & Barr, 2016  U.S.A. | 43 adolescents, 32.6% female (*M =* 15.4yr, *SD =* 2.4yr).  Recruited from small local community in southern U.S.  (Group allocation: NR) | Empirical :  Experimental study  -  2x2 mixed factorial design | To examine how VVGs influence young people, regarding hostility and stress. | Trait Aggression: measure trait aggression, acting control variable. (Buss & Warren, 2000)  SHS: report current mood (Anderson et al., 1995)  STAI: measure current anxiety (Spielberger et al., 1983) | Hostility results indicated a significant interaction between pre/post time and trait aggression, as well as main effect of time. No main effect for game condition or interaction with time. Bayes factor for hostility indicated moderately strong support for null hypothesis. Stress was found to have no significant effects for time or game condition. Small increase of stress levels in female players but not males due to VVGs. | Current evidence did not support beliefs that youth hostility increases due to VVGs but nor do they reduce stress. Suggestion that not stress that rose in females but annoyance at type of game, which were less likely to choose to play. |
| Ferguson & Donnellan, 2017  U.S.A. | 153 Italian adolescents (*M =* 16.8yr, *SD =* 1.2yr).  56.8% female.  Recruitment NR.  Neutral game (control), *N =* 51.  Violent-only game, *N =* 55.  Violent-sexist game, *N =* 48. | Empirical :  Experimental study  -  Confirmatory analysis test | Reanalysis of dataset to examine whether there is strong enough to support previous claim; a connection between “sexist” video games and  decreased empathy toward girls using an experimental  paradigm (Gabbiadini et al., 2016) | Empathy: how empathic participants felt towards a violent image with victim being female (Batson et al., 1987)  MRNI: assess core beliefs about masculinity and operationalise these (Levant et al., 2010).  Embodied presence scale: measure avatar identification (Van Looy et al., 2012). | No direct impact on empathy towards girls. Effect of game content on masculine beliefs was contingent but the effect was small and nearing insignificance. The experimental groups differed significantly and  substantially in terms of age suggesting that there might have been issues with the procedures used to randomly assign participants to conditions. | There is little evidence that suggested a link between sexism in games and real life. However, it is possible that a violent image of a “real” human may not manifest the same emotional response as a violent image in a game similar to VVGs. Current re-analysis highlights benefit of preregistration of studies, greater focus on unreliability of original results than evidence of sexism. |
| Fisher 2015  U.S.A. | 6 U.S. video game magazines (3 print, 3 online); review of 266 online and 142 physical articles, to establish representation of each top 10-bestselling games for both playable male and female protagonist-avatars. | Theoretical : Review  –  In-depth analysis of character portrayals using theory of Hegemonic Masculinity | Review the modern portrayal of men and women in video game magazines | Presence of male/females perspectives in text describing the protagonist  Presence of male/females within images from games  Words/phrases used within text to describe gameplay regarding portrayal of male/female characters within the game and its audience  Words/phrases used within text to describe the protagonist and their narrative  Protagonist interactions with NPCs and whether NPCs are male/female, and their portrayals | Women commonly represented as sex objects, focusing on physical characteristics. Women were less represented or considered secondary within magazines. The attitude that men are the ‘‘real gamers’’ was presented by online readers as they posted comments about the reviews or previews of various games. Overall, the praises of women presented in the magazines were few and faint. | Women are either portrayed as sex objects or ignored in favour of portrayals of men. Positive portrayals were few and weak in presentation. Within the magazines, female characters were used to grab attention but not expanded as actual characters of game play. May limit the acceptability of video games to women, as they are not viewed as equal in these images. |
| Fox & Tang, 2016  U.S.A. | 293 female adults (*M =* 26.2yrs, *SD =* 6.4yr).  Recruited through online forums, blogs and social media across multiple countries, most commonly U.S.A. and U.K. | Empirical : Survey  -  Cross-sectional online survey | To assess what coping strategies women use to prevent or mitigate harassment. | Video Game Harassment Scale: considers verbal and gameplay harassment (Fox and Tang, 2013)  Rumination about an Interpersonal Offense  Scale: to assess rumination about harassment within game (Wade et al., 2008)  Organizational  Unresponsiveness to Sexual Harassment questionnaire: perceptions of organisational responsiveness to harassment within game (Miner-Rubino and Cortina, 2007)  Withdrawal: developed to cover withdrawal specific to gaming context  Coping strategies: assess how women coped with harassment before, during and after gameplay. (Fitzgerald, 1990; Fox and Tang, 2013; Gutek and Koss, 1993; Gray, 2012b; Knapp et al., 1997). | Results supported the salience of toxic behaviour in online games and revealed that women’s experiences with harassment have a significant impact on their continued participation. Sexual harassment led to rumination, although general harassment did not. Both forms had adverse outcomes in influencing women’s play. | Women’s experiences with harassment can explain some sex/gender differences in gaming literature. Women may limit or avoid communication with other players to avoid harassment but many cooperative games need communication for quick coordination thereby reducing their ability to level up as quickly as male counterparts do. This could influence the visibility of female gamers as having skilled game play due to the self-imposed segregation. |
| Fraser, Elliott, de Bruin, Bherer & Dumoulin, 2014  CANADA | 23 women (*M =* 70.4yr, *SD =* 3.6yr).  Recruited through newspaper advertisements and senior organisations in Montreal. | Empirical : Experimental  -  Intervention pilot | To assess executive function and dual-task gait after a multicomponent intervention combining PFM training and VGD | Modified Stroop task: reading, colour denomination, inhibition and inhibition/switch conditions (Bohen et al., 1992)  TMT: indicator of EF abilities ( Sanchez-Cubillo et al., 2009; Spreen & Strauss, 1998; Srygley et al., 2009)  UI battery: measured the amount of urine leaked by weighing sanitary pads (Elliott et al., 2012) | Following training, the number of errors in inhibition/switch condition decreased significantly, TMT difference score improved marginally and number of back-errors during dual-task gait significantly decreased. Subgroup based on continence improvement revealed that only subjects who improved in pad test had significantly reduced numbers of back-errors during dual-task gait. | Suggestion that multicomponent intervention can improve executive functioning and dual-task gait of older women with mixed urinary incontinence. This could also provide a form of exercise to older women. |
| Gabbiadini, Riva, Andrighetto, Volpato & Bushman, 2016  ITALY | 154 high-school student volunteers (*M =* 16.8yr, *SD =* 1.2yr), 56.6% female.  Neutral game (control), *N =* 50.  Violent-only game, *N =* 55.  Violent-sexist game, *N =* 48. | Empirical : Experimental  -  Cross-sectional experiment using video game task | To investigate the short-term effects of playing violent-sexist video games on empathy for female violence victims. | Video game manipulation checks: identify game, excitement, sexualisation of females, frequently played video game  Player identification scale: how much participants identified with video game character (Van Looy et al., 2012)  MRNI-R: measures masculine beliefs (Levant et al., 2010)  Empathy: how empathic participants felt towards a violent image with victim being female (Batson et al., 1987) | Participants’ gender and their identification with violent male video game character moderated the effects of the exposure to sexist-VVGs on masculine beliefs. Violent-sexist video games increases masculine beliefs as male participants highly identified with the game character. Masculine beliefs negatively predicted empathic feelings for female violence victims. | Violent-sexist video games reduce empathy for female violence victims, in the short-term. Reduction in empathy partly occurs because video games increase masculine beliefs – tough, dominant, aggressive. Pronounced effects in male participants who strongly identified with misogynistic game characters. This could have a negative impact on women offline, either by males considering behaviour that is more violent to be acceptable or through sexist actions. |
| Gorbet & Sergio, 2018  CANADA | Study 1:  20 females,  10 AVG players (*M =* 26.5yr, *SD =* 7.7yr) and 10 who did not play, (*M =* 23.9yr, *SD =* 4.9yr).  Study 2:  20 females,  10 AVG players (*M =* 26.0yr, *SD =* 7.2yr) and 10 who did not play, (*M =* 24.9yr, *SD =* 6.9yr).  Both studies: Recruitment of participants using posters around York University campus. | Study 1:  Empirical : Experimental study  -  Neuroimaging experiment using 40 trials requiring fixation and touch targets during cue and delay periods within fMRI.  Study 2:  Empirical : Experimental study  –  Computer reaction time study across 40 trials using arm movement kinematics | Study 1:  To expand the knowledge of the effects of extensive action video game exposure on functional brain activity associated with visumotor processing. Examination of visuomotor-related brain activity in women with extensive history of AVG play.  Study 2:  To examine potential kinematic differences in arm movements made by women who play action video games and those who do not. | RT  MT  Standard condition: eyes and hand moved to same target  Non-standard condition: eyes and hand moved to 180^0^ difference directions for targets  Study 1:  fMRI: task used to study standard vs non-standard visuomotor performance  Study 2:  Touch screen computer: task used to study standard vs non-standard visuomotor performance | Study 1:  Total number of reaching errors or total errors did not significantly differ between AVG player and non-player groups. Similar errors made by both groups, moving prior to go signal, failing to initiate movement and some wrong direction movements. Mean reaction times not significantly different, nor were differences between two visuomotor mapping conditions for AVG and non-players. Statistically significant differences in fMRI showed non-gamer group had greater mean activity than AVG in left cuneus, left middle occipital gyrus, superior portion of the right posterior lobe of cerebellum.  Study 2:  AVG players had significantly faster reaction times, peak movement velocities, MT times and corrected MT related to non-players. Maximum velocity of movement was slightly significantly faster for standard condition to non-standard in both groups. AVG players had significantly larger ballistic and corrected variable errors than non-players. | AVGs suggested to evoke greater neural plasticity resulting in a variety of skill enhancements and decreased activity to reaction to stimulus. When there was no confinement of fMRI AVG players were shown to have faster RT and MT times. This could be due to the familiarity of movements which are needed in gaming. Compared to male gamers in a previous study, these results indicate different brain patterns of activity underlying visuomotor performance. Possible that AVGs may be biased in their design towards how the male audience responds to games over the smaller pool of female gamers. |
| Hartmann, Moller & Krause, 2015  THE NETHERLANDS | 444 students (*M =* 24.0yr, *SD =* 4.3yr), 137 females.  Recruited from Potsdam and Zurich universities. | Empirical : Experimental  -  Cross-sectional design using survey including video of video game | Examines the assumption that males play VVGs more because they anticipate more enjoyment and less guilt from engaging in virtual violence than females. | VVG use: frequently they play each genre of game using level of violence score (Krahé and Möller, 2010)  State Shame and Guilt Scale: assess anticipated guilt (Marschall et al., 1994)  Toronto Empathy Questionnaire: assess trait empathy (Spreng et al., 2009)  Mechanisms of Moral Disengagement Questionnaire: general tendency to justify physical violence (Bandura et al., 1996)  Video Game Needs Questionnaire: assess needs for sensation and aggression in video game play (Möller et al., 2010) | Men reported use of VVGs more often than women, and had higher scores for gratification, moral justification and anticipated enjoyment. Women had higher trait empathy and anticipated guilt. Gender significantly predicted VVG used via anticipated enjoyment and guilt. Gender significantly predicted use via empathy and anticipated guilt. Suggestion males use VVGs more frequently because they anticipated greater enjoyment since they have stronger tendency to justify physical violence. | Anticipated enjoyment and guilt uniquely affect exposure to VVGs. Users’ expectations of moral emotions like shame, moral disgust and pride may be particularly relevant in explaining limited female exposure to VVGs. Important role of trait empathy, females feel more guilt than males, thus play less frequently. Enjoyment users expect from virtual violence relates to moral justification and need to experience aggressive states in video games, seen more in men, suggests that these traits and anticipated emotions influence whether to play a game. |
| Harvey & Fisher, 2015  CANADA | 3 females, in prominent positions in games journalism and development. | Theoretical : Review  -  Commentary on female designers of games | Considers the context of high-profile women in digital games culture, focusing on relationship between gender-based action and feminist thought. | Gender in Industry: history and position of women working in digital game development  DEI: Canadian intervention related to diversity and feminist action  Responses to gender-based shifts in games journalism and development: Robin Hunicke’s talk, article by Leigh Alexander and Mare Sheppard’s presentation. | Female designers receive attention for reasons such as physical appearance, or being considered the token female. Tension building due to constant visibility of gender as designer, and not being recognised for work outside of gender. Issues from within with actions female designers taken not always showing support for inclusivity of taking on more women. | Due to the challenges which underpin being a visible woman in game culture, a more hostile environment has been created by men and often by women. This can act as a deterrent for women to access this line of work. Necessary for a shift from just women in gaming to feminists in gaming, and acknowledgements of skills over just gender. |
| Jansz & Martis, 2007  THE NETHERLANDS | 12 contemporary video games; 22 characters. Selected based on criteria; gender and race diversity of cast, narrative game play, available on PS2 or X-Box. | Empirical : Qualitative study –  Content analysis of introductory files to video games | Examines representation of characters in contemporary video games with respect to gender and race, and differences between earlier video games. | Gender: decision of characters sex made on physical features  Race: decision of race made on physical and verbal features  Role and position: distinguishing between 8 roles of characters (Beasley and Standley, 2002; Propp, 1968)  Appearance: examine stereotypes of appearance; attire, body, build, breasts, buttocks (Beasley & Standley, 2002; Downs  & Smith, 2005; Gauntlett, 2002) | Dominance of males in games (13/22). Majority of characters were white (15/22). Among leading characters there was equal gender distribution, but supporting characters were more often male (70%). Leading roles held dominant positions; there was no difference between gender. Female support characters held either dominant or equal positions to males, however 43% of male support characters were submissive; helper or friend. Sexy attire was mainly worn by female characters. Large draw on body type for both men and women; muscular or being thin with large breasts/buttocks. | Given the abundance of hyper-muscular men  and hypersexualized women in the video games, understandable that games may appeal to men more than women. This could be related to the objectification of women in video games. Suggestion that gender stereotyping is an important reason why young women play less than men. However, the central character being female could act as empowering female gamers. |
| Kasumovic, Blake, Dixson, & Denson, 2015  AUSTRALIA | Study 1:  467 participants (*M =* 30.9yr, *SD =* 8.5yr), 158 women.  Study 2:  462 adults (*M =* 30.7yr, *SD =* 9.1yr), 161 women.  Both studies:  Recruited via survey posting on Amazon’s Mechanical Turk Sept1-2^nd^ 2014, all from U.S. | Empirical :  Survey  –  Online cross-sectional survey | Study 1:  Examine differences of gender with the relationship between VVG play and self-ratings of own mate value and sexual interest, and whether repeated play of violent competitive games increase feelings of dominance.  Study 2:  To identify conscious mating-related motivations for playing VVGs and does this mediate interaction between gender and mate value. | Status-related variables: level of education, current occupation, current earning, expected future earnings, intelligence (Strenze, 2007), trait levels of dominance (Fabiansson, 2013)  General Social Survey Wordsum Vocabulary Test: assess understand of words (Cor, Haertel, Krosnick & Malhotra, 2012)  Mating-related variables: current relationship status, length of relationship, age of partner, whether they had children  Mate Value Scale: measure perceived desirability (Edlund & Sagarin, 2014)  SOI: assess attitudes towards sexuality (Penke & Asendorpf, 2008)  Study 1:  CTS: assess levels of intimate partner violence (Straus, Hamby, Boney-McCoy & Sugarman, 1996) | Study 1:  Men spent more time playing VVGs than women. Exposure distributions overlap – women players spent a large proportion of game time playing violent compared to non-violent. There was a significant positive relationship between sexual interest and VVG exposure. Higher education related to playing fewer VVGs. Significant effect between mate value and exposure, women that played more VVGs reported higher mate value than women who didn’t – not replicated in men. Women with more sons played more VVGs than those with fewer, no interaction between daughters.  Study 2:  Men reported higher levels of game exposure than women, overlap in exposure to violent games. Participants higher in sexual interest reported playing more VVGs. Significant positive association between mate value and exposure, acting as significant interaction between gender and mate value. Significant gender by mating motivation interaction with women and not men. Mediation analyses shows playing VVGs manifests as higher sense of mate value for women. | VVGs can have an interesting relationship with how women perceive themselves as partners within relationships; this could be related to wanting to share an interest with male counterparts to appear as a more attractive mate. Results suggest drive to play VVGs strongly associated with sexual perceptions. Alternatively having more sons could cause video game exposure due to social bonding mechanisms between mother and child. Suggestion that there are cognitive benefits to playing VVG exposure and greater wins in competitive scenario improves perceptions of an individual’s ability and self-esteem through self-serving bias. |
| Kaye, Gresty & Stubbs-Ennis, 2017  U.K. | Study 1:  489 online gamers (*M =* 25.0yr, *SD =* 6.1yr), 58.6% female.  Recruited through relevant gaming forums.  Study 2:  193 male players (*M =* 25.2yr, *SD =* 8.3yr)  Regular MMO players, *N =* 112 (30+ hours/week).  Regular FPS players, *N =* 81 (1-5 hours/week).  (Recruitment and location NR) | Empirical : Experimental  -  Within-subjects experimental design | Study 1:  To identify if gender swamping impacts upon competence perceptions towards female players.  Study 2:  To explore whether male social identity is related to sexist attitudes towards female players and whether this varies by gaming context (MMO vs FPS) | Study 1:  Competence perceptions: vignettes of fictitious players, rated with 5-point scale  Study 2:  Group Identification Measure: measure affiliation with male social identity (Doosje et al., 1995)  Gender Role Beliefs Scale: measure traditional gender-role beliefs (Brown & Gladstone, 2012; Kerr & Holden, 1996)  Video Game Sexism Scale: measure sexist beliefs in respective gaming context (Fox & Tang, 2014) | Study 1:  Participants gave significantly higher ratings of competence when female depicted as male avatar. Male players were not deemed more or less competent on their avatar gender.  Study 2:  No differences were found between MMOs and FPSs on social identity, gender-role beliefs and sexist attitudes in gaming. Gender-role beliefs significantly predicted sexist beliefs but not social identity for both FPS and MMOs. | Avatar gender within games has an effect on perceived player competence, with male avatars being considered more competent, particularly when played by females. This is possibly linked to men being considered more skilled within games, which could be related to general gender-role beliefs. Suggest use of non-gender avatars to avoid attribution of gender. |
| Linderoth & Öhrn, 2014  SWEDEN | 9 student online gamers (19-22yr, *M/SD =* NR), 4 female.  Recruited from larger questionnaire at University of Gothenburg and 1 active request to participate. | Empirical : Qualitative study  -  Thematic analysis of case study | Explore kinds of gender positions found in contemporary game culture and how this is experienced | Unstructured interviews: informal conversations focusing on games and gaming culture. | Assumption of players to be male, and therefore more competent. Interactions were notably different when representation of gender is female; dirtier or more polite. Female received more help or favours based on gender. Sexual harassment or expectations due to gender; coping strategies used were not using verbal communication or band with other female players. Anonymity that is offered suggests flexibility of gender for advantage or enjoyment, and real gender is taken less of a reference point. | Being a female gamer tends to be associated with being treated differently by other gamers. This can be beneficial but can also lead to being less recognised as a skilled player or harassment. Anonymity and these benefits offer some flexibility as players swap genders but experiences can be linked to having to “prove” their gender, and that is inflexible compared to age or nationality when considering the non-virtual identity. |
| Lynch, Tompkins, va Driel & Fritz, 2016  U.S.A. | 571 video games with playable female protagonists.  Selected from video game information taken from IGN, GiantBomb and Wikipedia, from 1983-2014. Excluding games featuring non-anthropomorphised characters, characters from pre-existing media franchises, and erotic video games. Randomly sampled 20 titles per year. | Empirical : Qualitative study  -  Content analysis of female characters in video games | Examine portrayal of female characters within games. Whether sexualisation of playable characters changes over time, whether this differs between game genre or ratings, the influence of sex on capability and role of female character. | Video game variables: critical score from professional critic, year of release, ESRB rating, genre and primary character.  Character variables: chest, buttock, wait leg, sexualisation index (Downs & Smith, 2010), physically capable, violently portrayed (Smith et al., 2003) | Sexualisation of female characters changed over time, with a lower values in time block 1 and 4 than 2 and 3. Fighting games featured most sexualised characters. Ratings which were for T or M audiences featured more sexualised characters. Recent female characters were more likely to be secondary than primary, and secondary characters are more sexualised. Sexualisation was positively correlated with capability. There was not a relationship between critical success of the games related to sexualisation of female characters. | Sexualisation of female characters is not shown to link to success of the game. It might be worthwhile investigating this interaction when considering game design, as high correlation between T- and M-rated games and sexualised characters. The use of women as secondary, sexualised characters may have a negative impact on how women are considered within real-life context, particularly by T-audiences. |
| Martey, Stromer-Galley, Banks, Wu & Consalvo, 2014  U.S.A. | 375 participants (*M =* ~29yr, *SD =* NR), 43.5% women.  Recruited through advertising on Facebook and WoW player forums.  Randomised into 110 groups of 3-4 players for task of 1.5hours. | Empirical : Experimental  -  Mixed methods design using survey and observational information | To examine how movement ad appearance behaviours differed by gender and by men who do or do not use female avatars | Chat variables: action-directives, polite phrases, appreciation phrases, emotional phrases, exclamation points, smile emoticons.  Avatar movement: move, clustering, jump  Avatar appearance: avatar gender, traditional hairstyle  Bem Sex Role Inventory: identifies alignment with social constructed gender roles (Allen & Core, 1997; Bem, 1974) | Avatar and player gender strong correlation, and gender-switching correlated with men being more likely to switch than women. Men did not seek to mask offline gender when using female avatar, but reinforced idealised notions of feminine appearance and communication. Men and women had significantly different player behaviours; action directive statements more common among men, all other chat variables more common in women, all movement variables were greater in men. | Utility of switching player gender is different between men and women, with men seeking beneficial behaviours that are offered to women online; however men are less likely to mask their gender switch than women, who may be using this to avoid online harassment. However, gender-switching does not include integrating the chosen gender into play-style. Possibility that avatar gender less matter of identity but strategic selection. |
| Martins, Williams, Harrison & Ratan, 2009  U.S.A. | 150 video games (368 characters). Sampled from The NPD Group sales data; Mar2005-Feb2006 for 9 major gaming systems; X-Box 360, X-Box, PS2, PS, Nintendo Gamecube, PSP, Nintendo GBA, DS and PC. | Empirical : Qualitative study -  Content analysis of female bodies within video games | Examine representations of female bodies; reflect realism, body proportions, interaction between power and body proportions, influence on game rating. | Difficultly setting: assessed by expert player from 30 minutes of play  Body proportion: height, head, chest, waist and hip widths (Norton et al., 1996)  Realism: codes rated on detail and pixilation  Rating and genre: ESRB ratings  Anthropometric Comparison sample: CAESER (Harrison and Robinette, 1998) | Distinct differences in way females were portrayed in video games, particularly in differing levels of photorealism and game rating. Female video game characters were at the highest level of photorealism and in games for children were thinner. Less realistic or games for adults featured large characters, but still had smaller chest, waists and hips than average American, with larger heads. | Video game culture emphasises thin bodies with unobtainable body proportion ratios for women. This may activate body dissatisfaction in female gamers and influence desire for such appearance in partners within male gamers. Due to the early age at which children play video games, it is possible that this could negatively influence perceptions of healthy bodies when growing up. |
| Matthews, Lynch & Martins, 2016  U.S.A. | Study 1:  149 female students (18-40yrs, *M/SD =* NR).  Recruited from telecommunications courses at Indiana University.  (Group allocation: NR)  Study 2:  197 male students (18-40yr, *M/SD =* NR).  (Group allocation: NR)  Both studies: Recruited from telecommunication courses at Indiana University. | Empirical : Experimental  -  Cross-sectional experiment using video game task | Study 1:  To investigate how ideal and hyper-ideal body types within video games affect body image disturbance for female players.  Study 2:  To investigate how ideal and hyper-ideal body types within video games affect males’ body image disturbance. | BES: assess participants’ feelings for body parts (Franzoi & Shields, 1984)  Body discrepancy: perceived from body size: desired body (Stunkard et al., 1983)  Trait Social Comparison tendency: assess trait social comparison tendencies (Botta, 1999)  Game usage & BMI  Study 1:  EDI: assess body attitudes (Garner et al., 1985)  Study 2:  PAM – SMAQ: attitudes towards muscularity (Edwards & Launder, 2000) | Study 1:  Results indicated that video games featuring ideal bodies exhibited no significant effect on player’s body image dissatisfaction and general body attitudes. However, hyper-idealised game bodies caused body image dissatisfaction and attitudes to improve compared to ideal bodies, less frequently to control.  Study 2:  No single main effects of video game bodies on males was found. Interaction between low comparison tendencies and less body image dissatisfaction, and more positive body attitudes in comparison. Hyper-idealised bodies worsened body image dissatisfaction and body attitudes only in low trait social comparison men. | Findings of hyper-idealised game bodies positively affecting women’s body perceptions diverges from previous work. May be result of downward social comparison or empowerment by physical capabilities of avatars.  Possible that males in the hyper-ideal condition engaged in upward social comparison and desired to look like characters due to adherence to male fantasies. May be that low social comparers are less discriminant about models of comparison when engaging, so trait social comparison is less about selectivity rather it is the extent. |
| Mortensen, Kristensen, Brooks & Brooks, 2013  DENMARK | 15 women with FMS, only 7 completed trial (*M =* 49.3yr, *SD =* 4.2yr).  Recruited from Reumaclinic Denmark. | Empirical :  Experimental  -  Mixed methods intervention; 45-minute semi-structured interview and survey responses at baseline and post-intervention. Intervention: 5 consecutive sessions with Wii, PS3 Move, X-Box Kinect with observation by occupational therapist. | To explore the experience women with FMS had, using MCVGs and explore symptom severity indicators and performance of ADL | ToP: assessment of play experience ( Bundy et al., 2001; Harkness & Bundy, 2001; Hess & Bundy, 2003)  VAS: assessment of pain (Carville et al., 2008; Wewers & Lowe, 1990)  BFI: assessment of fatigue (Akkaya et al., 2013; Mendoza et al., 1999)  ADL-Q: assessment of ADL performance (Waehrens et al., 2012) | Participants stated that they were distracted from pain symptoms during MCVG play; benefits included fun, manageable exercise, slow face and familiarity. Reported increase strength and body awareness but no indication of improvement in symptom severity. | Recommendation of exercise for management of fibromyalgia syndrome despite people finding this counterintuitive due to pain exacerbation. This may influence adherence to an exercise program. MCVGs may offer temporary pain relief and fun low impact exercise for women with FMS. |
| Near, 2013  U.S.A. | 399 games. Purposive sample drawn from 6400 games with at least 10,000 sales in U.S. Random selection from those that met inclusion criteria; major home console, 2005-2010, ESRB rating T or M, full MetaCritic score. | Empirical : Qualitative study –  Content analysis of video game U.S. box art | Examine whether character images in video game art predicts success as game sale; in particular this relation with female characters. | Sales Count: number of game copies sold in US  Box art: character present (none, male, female), position of character, sexualisation of female, portrayal of female  Control variables: review score, year of release, console maker, publisher, ESRB rating | 42% showed male characters exclusively, 7% female alone. Female characters in about one third of games; 19% of all games central. Male characters central in 66% of all games. Of all women shown, 21% sexualised. Female characters alone or central on box art reduced sales. Among T and M games, sales highest in games with box art shown non-central, sexualised female. Presence of central male also associated with higher sales. | Sexualisation of female characters is associated with higher sales only when no female characters are central, suggesting stereotypical depictions of characters e.g. male hero. Reinforced by improved sales when male central, particularly in M-rated games, which are linked to stereotypical masculinity due to features labelled mature; violence, sex or drug references. Possible that sexualised female characters may put off female consumers. |
| Norris, 2004  U.S.A. | 430 women (*M/SD =* NR).  Recruited by advertising via female-focused chat rooms, message boards and email. | Empirical :  Survey  -  Cross-sectional online survey | To explore gender identity and aggressive personality in the context of computer use and games. | Perceived hostility of online environment: measures harassment and identification with online community  SRSS: measures attitudes towards women (Burt, 1980)  modified version of AIV: measures attitudes towards aggression as a means of compliance in relationships (Burt, 1980)  PAQ: measures gender identity (Spence & Helmreich, 1979)  Aggression Questionnaire: physical aggression, verbal aggression, anger and hostility (Buss & Perry, 1992; Carlson et al., 1989)  HOEQ: assesses online sexual harassment and friendliness scale. | No differences between women who played computer games without visiting chat rooms and vice versa with experiences of sexual harassment, but those who gamed experiences less friendship. No difference in sex role stereotyping between exclusive gaming or chatting. Those who chatted without gaming were more accepting of interpersonal violence. No difference in masculinity between chatters and gamers, but chatters found to be slightly more feminine. Gamers were not more aggressive when compared to chatters, but those that played more hours were more aggressive than those who played less. Women who were more masculine spent more hours on a computer than those that were feminine. | Computer-based tasks were considered to be a masculine activity, which may put some women off. It is possible that this impacts how computers are used and why those who gamed were less likely to experience friendships online. Suggestions that creating a non-violent computer opportunity would reinforce the idea that computer use was an androgynous activity. However, it is possible that this was related to an older sample of women, although this is not known due to a lack of information. |
| Olson, Kutner, Warner, Almerigi, Baer, Nicholi & Beresin, 2007  U.S.A. | 1254 7^th^ and 8^th^ grade students (*M* *=* NR, *SD =* NR), 53% female.  Recruited from English/Language Arts classes in 2 middles schools in Pennsylvania and South Carolina. | Empirical : Survey  -  Cross-sectional survey | To compare the video and computer game play patterns of young adolescent boys and girls | Game exposure and preferences: to assess violent exposure – asked to list 5 games played frequently over 6 months | Majority of children played some form of video game over 6 months (93.6%), 48.8% had at least on M-rated game as one of their most played (67.9% male: 29.2% female). Boys were ten times as likely as girls to play for over 15 hours a week. Children who had a gaming station in their room (18.2%) were twice as likely to play 15+ hours or M-rated games. Games used for fun, creative reasons and manage emotions. | M-rated game play could be risk marker for girls but for boys were linked with playing with friends. This could be related to the social gender identities related to how children communicate with each other. The use of violent games to cope with anger may be healthy or unhealthy depending on the child. |
| Paaßen, Morgenroth & Stratemeyer 2017  GERMANY | 10 games, across literature  (Sampling NR). | Theoretical : Review  -  Literature review | To investigate literature on gender and gaming to examine the male gamer stereotype in terms of accuracy, persistence, effects and future perspective. | Predominantly male gamers: assessed through time investment, self-identification, design elements and content, genre, skill  Male stereotype: relation to self-identification, social theory, visibility. | Prior belief that there are more male gamers, but considered to be more evenly distributed. Stereotype relatively unattractive. Time assessment of games investment was moderately accurate outside of extremes. Men tended to identify more as gamers, particularly when younger – might be result of marketing. Lower level of women played “hard-core” genre games, design elements thought to add to women being more casual gamers than men. Skill appears linked to time investment on games, if women spent as much time on games as men, negative stereotype less likely.  Visible players in gamer roles are mostly male – leads to association between being male and stereotype. | Many women play in the same way as men, thus male gamer stereotype has limited accuracy. Men are more likely to be visible, identifying as gamers. Video game culture is actively hostile towards women, limiting them to being either female or a gamer, therefore it is unsurprising that they are less visible. Suggest that if more women visible as gamers, culture is likely to change. Marginalisation of visible women in video game culture may lead to negative psychological outcomes – unwelcome, isolated, unappealing hobby. |
| Puente Bienvenido & Lase Diaz, 2015  SPAIN | *N =* 956 gamers in the Diablo III (Role Play Game) | Empirical: Mixed methods approach with a triangulation of participant observation online and interviews selected from a survey | To analyse how players and fans of Diablo III discuss gender choreographies  as forms of shared agencies (i.e., online game practices, digital devices, platforms, design and commercialisation shared by gamers from both genders) among players  and features of the game with the background of sexism and stereotypes | Online ethnography: inside the video game Diablo III.  Online survey In-depth interviews | Results show women access Diablo III through acquaintances (i.e., partner/boyfriend or family members/friends), while men do it by themselves. The usual motivation of female’s partners was they will play for them, a conflict usually solved stopping doing it, and buying a copy for themselves after discovering they like the game and want to play themselves.  Another result is women do not usually find content for women in the video game industry and still there are paternalistic and masculine mechanisms (i.e., sexist practices; particularly when anonymity plays a role). However, even with these issues, women who play Diablo III play longer and spend more money than males. | Suggests game practices may contribute  to constructing gender and destabilizing certain existing expectations. Female role players are not in the classic gamer profile yet, but companies are making efforts to change gender stereotypes in games (e.g., this should start with avatars, as women usually prefer feminine avatars with complex facets, such as capacities, play styles, a part of the appearance). |
| Shen, Ratan, Cai, & Leavitt, 2016  U.S.A. | Study 1:  9483 online players  82% men (*M =*  33.7yr, *SD =* 9.9yr)  18% women (*M =* 35.8yr, *SD =* 11.6yr).  Sampled by active play within one-week window.  (Location NR)  Study 2:  18,000 valid responses  74.5% men (*M =* 23.7yr, *SD =* NR)  25.5% women (*M =* 23.2yr, *SD =* NR)  2000 survey respondents with behavioural log data as well (23.3% female) | Empirical : Experimental  -  Server-collected longitudinal design | Study 1:  Test whether there are true gender differences using EQ2  Study 2:  Test whether there are true gender differences using CR3 | Gender: demographic gender from server log  Study 1:  Total play time: total time from server log  Performance: speed of character advancement (Steinkuehler & Duncan, 2008; Yee, 2001) against play time  Character class: 24 character classes  Account ID: considers multiple characters from same account  Guild ID : assesses guild membership, by advancement goals, play styles, resources and inner dynamics which influence individual performance (Ducheneaut et al., 2007; Williams et al., 2006)  Study 2:  New character level: captures each level up event (Ducheneaut, et al., 2006; Shen, 2014)  Time to level up: marginal rate of advancement  Character ID: information on players’ unique character ID.  Player Account ID: information on player’s unique account ID. | Study 1:  Men were younger than women, and played slightly more hours on average. Men on average reached higher character level than women, but no effect for skill. Interaction models showed that women advanced in the game at least as fast as men.  Study 2:  The more advanced the character, the more time needed to obtain another level. No gender effect. Interaction term of new level and gender was found to be significant. At level 15 women’s levelling advantage over men reversed. | Controlling for all factors, women who play as much as men reach similar levels; however, women tend to play less or stop early. Studies suggest gender stereotyping is false and potential cause for unequal participation. Research should not focus on play style or preference – consider context and conditions, e.g., peer community, gender expectations. This may encourage more equal participation between genders. |
| Song & Fox, 2016  U.S.A. | 174 female gamers (*M =* 20.1yr, *SD =* 2.6yr).  Recruited via Otomedream online forum, dedicated to female-oriented games in China. | Empirical : Survey  -  Cross-sectional survey | Examine influence of RVGs on beliefs and attitudes | Consumption of RVG: game play hours on average week  Identification with Avatar: measure identification with avatars (Cohen, 2001)  PSR with romantic target: reflection of nature of interaction with romantic targets in RVGs (Perse & Rubin, 1989)  Romantic beliefs: measure participants’ idealised romantic beliefs (Sprecher & Mett, 1989)  Covariates: age, consumption of romantic-specific media genres, general media consumption. | No significant direct relationship between RVG consumption and romantic beliefs. Playing RVGs positively related to identification with avatar. Identification not significantly related to idealised romantic beliefs but influenced by mediator of PSR. RVG play and PSRs with romantic targets not significant but identification may be mediator. Women with higher identification with avatars had stronger PSRs with romantic targets. Women with higher levels of PSRs had greater endorsement of idealised romantic beliefs. | RVGs could be related to the individual’s identity as high linked to identification with avatar. This suggests an internal process which mediates PSR and RVG play. Cognitive processes related to RVG exposure could influence how beliefs and attitudes are shaped. Possible that this type of game is more accessible to female audiences due to lack of violence, but has limited exposure within Western cultures. |
| Tian & Qian, 2014  CHINA | 98 primary school children (*M =* 16.2yr, *SD =* 1.6yr).  Gender NR.  Violent computer game group, *N* = 49.  Nonviolent computer game group, *N* = 49. | Empirical : Experimental  -  Multi-factor experimental design | Examine gender differences in aggression among children after playing violent computer games | Computer game: violent (Virtual COP2), non-violent (Fight Landlord) (Anderson & Bushman, 2001)  Goal Words: 50 aggressive: 50 non-aggressive | Significant main effect of goal word with reaction time, and reaction time of aggressive word significantly longer. Average aggressive priming score of girls no difference between games. However, for boys this was significantly higher for those playing violent games. | Significant gender differences in aggression among children following violent computer game, in favour of male children being more aggressive. Repeated exposure to violent media may form aggressive cognitive structure for boys, thus showing explicit aggressions. This may not appear as strongly in female gamers. |
| Tomkinson & Harper, 2015  U.S.A. | Commentary on a series of tweets concerning Felicia Day, between Ryan Perez and Wil Wheaton. (Number of tweets NR) | Empirical : Qualitative  -  Review of Twitter incident | Analyse details of Twitter incident between Perez and Day in context of feminist research; discuss whether position of women in game culture is changing | The Incident: series of tweets targeting Felicia Day as female gamer by journalist Ryan Perez and backlash from Wil Wheaton | Perez commented on Felicia Day not providing useful additions to gaming, reducing her to a sexual symbol representative of women in game culture and targeted her performance as a women in game culture. This was met with responses calling Perez out on sexism, and in particular that he is an “ignorant misogynist” by Wheaton. Perez fired from position at Destructoid journalism. | Increase of women in gaming culture challenges the masculine custodianship of this; however, there is growing resistance to misogynistic comments from members of the community. Incident between Perez and Day could function as crackdown on misogynistic tendencies and change of attitude due to Perez being broadly condemned. |
| Vermeulen, Castellar & Looy, 2014  BELGIUM | 39 female college students (*M =* 24.3yr, *SD =* 4.9yr).  Recruited via email and flyers distributed across Ghent University.  (Group allocations NR) | Empirical : Experimental  -  Multi-factor experimental design including game play | Investigate the effect of opponent gender on game experience of female players | Trait competitiveness: measured effect on emotions and perceived skill (Houston et al., 2002)  9-point SAM: measure affective reaction to certain stimulus; pleasure, arousal, dominance (Lang, 1980)  Objective performance: playing time, player’s score in losing condition, AI’s score | Winning was linked to higher pleasure, dominance, skill and lower challenge. More stress when playing against a male. Self-perceived skills and perceived opponent skills were modulated by trait competitiveness with larger effect size for low competitive women. | Women more likely to take gender as sign of gaming ability – stereotype threat theory suggests framing as this as artefact caused by threat of confirming a negative stereotype as self-characterisation and leave women misidentifying with games. |
| Vieira, 2014  U.S.A. | 145 girls *(M =* 11.3yr, *SD =* 2.0yr) across 12 countries.  Recruited by Club Pony Pals administrator over 4-week period. | Empirical : Survey  -  Cross-sectional online survey | Examine the effects of prosocial gaming on girl’s thoughts about perceived justified and unjustified aggressive attitudes | Prosocial play score: prosocial content and amount of playtime, rated by coding schema (Russell & Carroll, 1999)  Perspective-taking: 5 perspective-taking stories (Krcmar & Valkenburg, 1999; Krcmar & Vieira, 2005; Vieira & Krcmar, 2011)  Sympathy: assess sympathy (Vieira & Krcmar, 2011)  MIIV scale: measure moral reasoning about violence (Krcmar & Valkenburg, 1999; Krcmar & Vieira, 2005; Vieira & Krcmar, 2011) | Strong relationship between prosocial gaming exposure and level of perspective-taking. Perspective-taking related to level of sympathy, suggest mediation between prosocial gaming exposure and sympathy. Those who sympathised more considered less severe and severe justified violence as wrong, significant path from perspective-taking to severe unjustified scenario. Justification for violence was considered but not supported. Positive relationship between age and perspective-taking ability not supported. | Suggests that relationship between prosocial video gaming and assessments about different violent scenarios is mediated by differences in perspective-taking and sympathy. Younger children may feel sorry for others, without imaging what they are accuracy going through. Forums offered by games may encourage greater involvement with game and community. |
| Warden, Stanworth & Chang, 2016  TAIWAN | 348 undergraduates (*M =* NR, *SD =* NR), 246 females.  Recruited from an undergraduate class on business negotiation. | Empirical : Survey  -  Observation of virtual classroom use across 5 sessions followed by cross-sectional survey | Examine degree to which gaming experience and gender influence sense of presence in virtual world learning classroom | Telepresence scale: feelings of presence in virtual settings (Kim & Biocca, 1997; Klein, 2003; Nelson, Yaros, & Keum, 2006; Persky et al., 2009)  Control variables: gender, gaming experience, gamer, software ownership | Feelings of presence in virtual class did not differ significantly across gender. Purchasing and owning software did not impact presence. Relationship between higher frequency of gaming and higher levels of presence. | Gender and previous software experience do not significantly disadvantage learners from experiencing benefits of virtual classroom. Leaners with less previous gaming experience virtual classrooms similarly to gamers. Just as likely that women will success in this environment as well as men. |
| Weybright, Dattilo & Rusch, 2010  U.S.A. | 2 women over (86-93yr).  Recruited from local community assisted living facility (Pennsylvania) | Empirical :  Experiment  -  Single-subject multiple baseline ABAB designed intervention | Examine effects of an interactive video game on attention to task and positive affect of older adult women with mild cognitive impairment | MMSE: measure cognitive impairment (Folstein et al., 1975; Kurlowicz & Wallace, 1999)  Positive affect: facial response (Ekman & Friesen, 1975) and frequency of response (Briton & Hall, 1995)  Attention: fixated eye gaze observed | Participants reported enjoyment of game.  Increased averages for attention to task and positive affect. Increased ability to recall routine events and scheduling around bowling trials in 1 participant. | Demonstrated cognitive benefits of participating in video games. Opportunity to participate in engaging, stimulating and self-directed intervention activity at low-cost. Possibly to take intervention further and compare responses within male and female populations. |
| Yang, Huesmann & Bushman, 2014  U.S.A. | 242 university students (*M =* NR, *SD =* NR), 66% women.  Voluntary participation for credit.  (Group allocation NR) | Empirical : Experimental  –  2x2 between subjects design with video game priming | Examine the effects of whether avatar gender in VVGs prime aggression | Aggression: amount of hot sauce participant chose for “partner” (Lieberman, Solomon, Greenberg, & McGregor, 1999)  Control variables: frequency of game play, competitiveness, identification with game avatars | Male behaved more aggressively than females. Participants who played as a male avatar behaved more aggressively; both male and female. Approaching significance for interaction between avatar and participant genders. | Playing as a male avatar increased violence in participants. This may be related stereotyped masculine beliefs, particularly as male participants were more likely to identify with male violent male avatars. Repeat priming from aggressive, stereotyped avatars more susceptible to displaying long-term aggressive traits and behaviour within males. |

Note: Acronyms: Acceptance of Interpersonal Violence (AIV), Action Video Game (AVG), Attitudes toward Women Scale (AWS), Body esteem scale (BES), Brief Fatigue inventory (BFI), Chevaliers’ Romance III (CR3), Civilian American and European Surface Anthropometry Resource (CAESER), Conflict Tactics Scale (CTS), Computer Player Unit (CPU), Daily Living Activities (ADL), Daily Living Activities Questionnaire (ADL-Q), Deployment of Quality Function House (FQD), Difference Engine Initiative (DEI), Eating Disorder Inventory (EDI), Executive Functioning (EF), Entertainment Software Rating Board (ESRB), EverQuest II (EQ2), Fibromyalgia Syndrome (FMS), First Person Shooters (FPSs), Functional Magnetic Resonance Imaging (fMRI), Gameboy Advance (GBA), Hostile Online Environment Questionnaire (HOEQ) , IGN Entertainment (IGN), Immersive Virtual Environments (IVE), Magnetic Resonance Imaging (MRI), Male Role Norms Inventory (MRNI), Male Role Norms Inventory (MRNI-R), Massively Multiplayer Online (MMO), Mature (M), Mini-Mental State Examination (MMSE), Moral Interpretation of Interpersonal Violence (MIIV), Motion-Controlled Video Games (MCVGs), Movement Times (MT), Nintendo Dual Screen (DS), Non Playable Characters (NPCs), Not reported (NR), Pan-European Game Information code (PEGI), Parasocial relationships (PSR), Pelvic Floor Muscle (PFM), Personal Attributes Questionnaire (PAQ), PlayStation (PS), PlayStation 2 (PS2), PlayStation 3 (PS3), PlayStation Portable (PSP), Positive Attitudes of Muscularity (PAM), Presence Questionnaire (PQ), Quality Function Deployment (QFD), Reaction Times (RT), Revised Sociosexuality Inventory (SOI), Romantic Video Game (RVGs), Self-Assessment Manikin (SAM), Self-Efficacy in Technology and Science (SETS), Sex Role Stereotyping Scale (SRSS), State Hostility Scale (SHS), State/Trait Anxiety Inventory (STAI), Swansea Muscularity Attitudes Questionnaire (SMAQ), Taylor Competitive Reaction Time task (TCRT), Teen (T), Test of Playfulness (ToP), Trail Making Test (TMT), United Kingdom (U.K.), United States (U.S.), United States of America (U.S.A), Urinary Incontinence (UI), User-Centred Design (UCD), Video Game Dancing (VGD), Violent Video Games (VVGs), Visual Analogue Scale (VAS), World of Warcraft (WoW)

References in APA:

Anderson, C. A., & Murphy, C. R. (2003). VVGS and aggressive behaviour in young women. *Aggressive behaviour*, *29*(5), 423-429. https://doi.org/10.1002/ab.10042

Barlett, C. P., & Harris, R. J. (2008). The impact of body emphasizing video games on body image concerns in men and women. *Sex Roles*, *59*(7-8), 586-601. http://dx.doi.org/10.1007/s11199-008-9457-8

Beck, V. S., Boys, S., Rose, C., & Beck, E. (2012). Violence against women in video games: A prequel or sequel to rape myth acceptance?. *Journal of interpersonal violence*, *27*(15), 3016-3031. https://doi.org/10.1177/0886260512441078

Behm-Morawitz, E., & Mastro, D. (2009). The effects of the sexualisation of female video game characters on gender stereotyping and female self-concept. *Sex roles*, *61*(11-12), 808-823. http://dx.doi.org/10.1007/s11199-009-9683-8

Bergey, B. W., Ketelhut, D. J., Liang, S., Natarajan, U., & Karakus, M. (2015). Scientific inquiry self-efficacy and computer game self-efficacy as predictors and outcomes of middle school boys’ and girls’ performance in a science assessment in a virtual environment. *Journal of Science education and Technology*, *24*(5), 696-708. https://doi.org/10.1007/s10956-015-9558-4

Blumberg, F. C., & Sokol, L. M. (2004). Boys' and girls' use of cognitive strategy when learning to play video games. *The Journal of General Psychology*, *131*(2), 151-158. https://doi.org/10.3200/GENP.131.2.151-158

Burgess, M. C., Stermer, S. P., & Burgess, S. R. (2007). Sex, lies, and video games: The portrayal of male and female characters on video game covers. *Sex roles*, *57*(5-6), 419-433. https://doi.org/10.1007/s11199-007-9250-0

Carrasco, Á. E. (2016). Acceptability of an adventure video game in the treatment of female adolescents with symptoms of depression. *Research in Psychotherapy: Psychopathology, Process and Outcome*, *19*(1). https://doi.org/10.4081/ripppo.2016.182

Castillo, N. G., & Doral, T. B. (2016). Women’s image on video game covers: A comparative analysis of the Spanish market (2011-2015). *Prisma Social*, (1), 120-155. <http://revistaprismasocial.es/article/view/1316/1380>

Chou, W. H. (2011). An empirical research on female stereotyping in video game design. In *Proceedings of International Conference on Environmental Science and Technology (ICEST 2011)*. http://www.ipcbee.com/vol6/no1/84-F10035.pdf

Cortés-Picazo, L. C. (2016). Transgression of traditional gender identities through the graphical representation of women protagonists video game developed by children. *Arte, Individuo y Sociedad*, *28*(3), 459-473. http://dx.doi.org/10.5209/rev_ARIS.2013.v28.n3.48951

Cote, A. C. (2017). “I Can Defend Myself” Women’s Strategies for Coping With Harassment While Gaming Online. *Games and Culture*, *12*(2), 136-155. https://doi.org/10.1177/1555412015587603

Coyne, S. M., Padilla-Walker, L. M., Stockdale, L., & Day, R. D. (2011). Game on… girls: Associations between co-playing video games and adolescent behavioural and family outcomes. *Journal of Adolescent Health*, *49*(2), 160-165. https://doi.org/10.1016/j.jadohealth.2010.11.249

Crowe, N., & Watts, M. (2014). ‘When I click “ok” I become Sassy–I become a girl’. Young people and gender identity: subverting the ‘body’ in massively multi-player online role-playing games. *International Journal of Adolescence and Youth*, *19*(2), 217-231. https://doi.org/10.1080/02673843.2012.736868

DeNoyelles, A., & Seo, K. K. J. (2012). Inspiring equal contribution and opportunity in a 3d multi-user virtual environment: Bringing together men gamers and women non-gamers in Second Life®. *Computers & Education*, *58*(1), 21-29. https://doi.org/10.1016/j.compedu.2011.07.007

Driesmans, K., Vandenbosch, L., & Eggermont, S. (2015). Playing a videogame with a sexualized female character increases adolescents' rape myth acceptance and tolerance toward sexual harassment. *Games for health journal*, *4*(2), 91-94. https://doi.org/10.1089/g4h.2014.0055

Eastin, M. S. (2006). Video game violence and the female game player: self‐and opponent gender effects on presence and aggressive thoughts. *Human Communication Research*, *32*(3), 351-372. https://doi.org/10.1111/j.1468-2958.2006.00279.x

Ferguson, C. J., Trigani, B., Pilato, S., Miller, S., Foley, K., & Barr, H. (2016). VVGS don’t increase hostility in teens, but they do stress girls out. *Psychiatric quarterly*, *87*(1), 49-56. https://doi.org/10.1007/s11126-015-9361-7

Ferguson, C. J., & Donnellan, M. B. (2017). Are associations between “sexist” video games and decreased empathy toward women robust? A reanalysis of Gabbiadini et al. 2016. *Journal of youth and adolescence*, *46*(12), 2446-2459. https://doi.org/10.1007/s10964-017-0700-x

Fisher, H. D. (2015). Sexy, dangerous—and ignored: An in-depth review of the representation of women in select video game magazines. *Games and culture*, *10*(6), 551-570. https://doi.org/10.1177/1555412014566234

Fox, J., & Tang, W. Y. (2017). Women’s experiences with general and sexual harassment in online video games: Rumination, organizational responsiveness, withdrawal, and coping strategies. *New Media & Society*, *19*(8), 1290-1307. https://doi.org/10.1177/1461444816635778

Fraser, S. A., Elliott, V., de Bruin, E. D., Bherer, L., & Dumoulin, C. (2014). The effects of combining videogame dancing and pelvic floor training to improve dual-task gait and cognition in women with mixed-urinary incontinence. *GAMES FOR HEALTH: Research, Development, and Clinical Applications*, *3*(3), 172-178. https://doi.org/10.1089/g4h.2013.0095

Gabbiadini, A., Riva, P., Andrighetto, L., Volpato, C., & Bushman, B. J. (2016). Acting like a tough guy: Violent-sexist video games, identification with game characters, masculine beliefs, & empathy for female violence victims. *PLoS one*, *11*(4), e0152121. https://doi.org/10.1371/journal.pone.0152121

Gorbet, D. J., & Sergio, L. E. (2018). Move faster, think later: Women who play action video games have quicker visually-guided responses with later onset visuomotor-related brain activity. *PloS one*, *13*(1), e0189110. https://doi.org/10.1371/journal.pone.0189110

Hartmann, T., Möller, I., & Krause, C. (2015). Factors underlying male and female use of VVGS. *New Media & Society*, *17*(11), 1777-1794. https://doi.org/10.1177/1461444814533067

Harvey, A., & Fisher, S. (2015). “Everyone Can Make Games!”: The post-feminist context of women in digital game production. *Feminist Media Studies*, *15*(4), 576-592. https://doi.org/10.1080/14680777.2014.958867

Jansz, J., & Martis, R. G. (2007). The Lara phenomenon: Powerful female characters in video games. *Sex roles*, *56*(3-4), 141-148. https://doi.org/10.1007/s11199-006-9158-0

Kasumovic, M. M., Blake, K., Dixson, B. J., & Denson, T. F. (2015). Why do people play VVGS? Demographic, status-related, and mating-related correlates in men and women. *Personality and Individual Differences*, *86*, 204-211. https://doi.org/10.1016/j.paid.2015.06.018

Kaye, L. K., Gresty, C. E., & Stubbs-Ennis, N. (2017). Exploring stereotypical perceptions of female players in digital gaming contexts. *Cyberpsychology, Behaviour, and Social Networking*, *20*(12), 740-745. https://doi.org/10.1089/cyber.2017.0294

Linderoth, J., & Öhrn, E. (2014). Chivalry, subordination and courtship culture: Being a ‘Woman’ in online games. *Journal of Gaming & Virtual Worlds*, *6*(1), 33-47. https://doi.org/10.1386/jgvw.6.1.33_1

Lynch, T., Tompkins, J. E., van Driel, I. I., & Fritz, N. (2016). Sexy, strong, and secondary: A content analysis of female characters in video games across 31 years. *Journal of Communication*, *66*(4), 564-584. https://doi.org/10.1111/jcom.12237

Martey, R. M., Stromer-Galley, J., Banks, J., Wu, J., & Consalvo, M. (2014). The strategic female: gender-switching and player behaviour in online games. *Information, Communication & Society*, *17*(3), 286-300. https://doi.org/10.1080/1369118X.2013.874493

Martins, N., Williams, D. C., Harrison, K., & Ratan, R. A. (2009). A content analysis of female body imagery in video games. *Sex roles*, *61*(11-12), 824. https://doi.org/10.1007/s11199-009-9682-9

Matthews, N. L., Lynch, T., & Martins, N. (2016). Real ideal: Investigating how ideal and hyper-ideal video game bodies affect men and women. *Computers in Human Behaviour*, *59*, 155-164. https://doi.org/10.1016/j.chb.2016.01.026

Mortensen, J., Kristensen, L. Q., Brooks, E. P., & Brooks, A. L. (2015). Women with fibromyalgia’s experience with three motion-controlled video game consoles and indicators of symptom severity and performance of activities of daily living. *Disability and Rehabilitation: Assistive Technology*, *10*(1), 61-66. https://doi.org/10.3109/17483107.2013.836687

Near, C. E. (2013). Selling gender: Associations of box art representation of female characters with sales for teen-and mature-rated video games. *Sex roles*, *68*(3-4), 252-269. https://doi.org/10.1007/s11199-012-0231-6

Norris, K. O. (2004). Gender stereotypes, aggression, and computer games: An online survey of women. *Cyberpsychology & Behaviour*, *7*(6), 714-727. https://doi.org/10.1089/cpb.2004.7.714

Olson, C. K., Kutner, L. A., Warner, D. E., Almerigi, J. B., Baer, L., Nicholi, A. M., & Beresin, E. V. (2007). Factors correlated with VVG use by adolescent boys and girls. *Journal of adolescent health*, *41*(1), 77-83. https://doi.org/10.1016/j.jadohealth.2007.01.001

Paaßen, B., Morgenroth, T., & Stratemeyer, M. (2017). What is a true gamer? The male gamer stereotype and the marginalization of women in video game culture. *Sex Roles*, *76*(7-8), 421-435. https://doi.org/10.1007/s11199-016-0678-y

Puente Bienvenido, H., & Lasen Diaz, A. (2015). Gender choreographies in online game spaces. Female players, fans and gender conflicts in video games. *REDES COM-REVISTA DE ESTUDIOS PARA EL DESARROLLO SOCIAL DE LA COMUNICACION*, (11), 155-183. http://revista-redes.hospedagemdesites.ws/index.php/revista-redes/article/view/369/407

Shen, C., Ratan, R., Cai, Y. D., & Leavitt, A. (2016). Do men advance faster than women? Debunking the gender performance gap in two massively multiplayer online games. *Journal of Computer-Mediated Communication*, *21*(4), 312-329. https://doi.org/10.1111/jcc4.12159

Song, W., & Fox, J. (2016). Playing for Love in a Romantic Video Game: Avatar Identification, Parasocial Relationships, and Chinese Women's Romantic Beliefs. *Mass Communication and Society*, *19*(2), 197-215. https://doi.org/10.1080/15205436.2015.1077972

Tian, J., & Qian, Z. (2014). Are boys more aggressive than girls after playing violent computer games online? An insight into an emotional Stroop task. *Psychology*, *5*(01), 27. http://dx.doi.org/10.4236/psych.2014.51006

Tomkinson, S., & Harper, T. (2015). The position of women in video game culture: Perez and Day's Twitter Incident. *Continuum*, *29*(4), 617-634. https://doi.org/10.1080/10304312.2015.1025362

Vermeulen, L., Núñez Castellar, E., & Van Looy, J. (2014). Challenging the other: Exploring the role of opponent gender in digital game competition for female players. *Cyberpsychology, Behaviour, and Social Networking*, *17*(5), 303-309. https://doi.org/10.1089/cyber.2013.0331

Vieira Jr, E. T. (2014). The relationships among girls’ prosocial video gaming, perspective-taking, sympathy, and thoughts about violence. *Communication Research*, *41*(7), 892-912. https://doi.org/10.1177/0093650212463049

Warden, C. A., Stanworth, J. O., & Chang, C. C. (2016). Leveling up: Are non-gamers and women disadvantaged in a virtual world classroom?. *Computers in Human Behaviour*, *65*, 210-219. https://doi.org/10.1016/j.chb.2016.07.033

Weybright, E. H., Dattilo, J., & Rusch, F. R. (2010). Effects of an interactive Video Game (Nintendo Wii (TM) on older women with mild cognitive impairment. *Therapeutic Recreation Journal*, *44*(4), 271. https://search.proquest.com/docview/921547910?pq-origsite=gscholar

Yang, G. S., Huesmann, L. R., & Bushman, B. J. (2014). Effects of playing a VVG as male versus female avatar on subsequent aggression in male and female players. *Aggressive behaviour*, *40*(6), 537-541. https://doi.org/10.1002/ab.21551
